# 3D-Printed Isoniazid Tablets for the Treatment and Prevention of Tuberculosis—Personalized Dosing and Drug Release

**DOI:** 10.1208/s12249-018-1233-7

**Published:** 2019-01-07

**Authors:** Heidi Öblom, Jiaxiang Zhang, Manjeet Pimparade, Isabell Speer, Maren Preis, Michael Repka, Niklas Sandler

**Affiliations:** 1 Pharmaceutical Sciences Laboratory, Åbo Akademi University, Artillerigatan 6A, 20520 Åbo, Finland; 2 Department of Pharmaceutics and Drug Delivery, The University of Mississippi, Oxford, Mississippi 38677 USA; 3 Institute of Pharmaceutics and Biopharmaceutics, Heinrich Heine University, Universitätsstr. 1, 40225 Düsseldorf, Germany; 4 Pii Center for Pharmaceutical Technology, The University of Mississippi, Oxford, Mississippi 38677 USA

**Keywords:** 3D printing, personalized medicine, hot-melt extrusion, immediate release, sustained release

## Abstract

The aim of the present work was to produce 3D-printed oral dosage forms with a sufficient drug dose displaying various release profiles. Hot-melt extrusion was utilized to produce drug-loaded feedstock material that was subsequently 3D-printed into 6, 8, and 10 × 2.5 mm tablets with 15% and 90% infill levels. The prepared formulations contained 30% (*w*/*w*) isoniazid in combination with one or multiple pharmaceutical polymers possessing suitable properties for oral drug delivery. Thirteen formulations were successfully hot-melt extruded of which eight had properties suitable for fused deposition modeling 3D printing. Formulations containing HPC were found to be superior regarding printability in this study. Filaments with a breaking distance below 1.5 mm were observed to be too brittle to be fed into the printer. In addition, filaments with high moisture uptake at high relative humidity generally failed to be printable. Different release profiles for the 3D-printed tablets were obtained as a result of using different polymers in the printed formulations. For 8 mm tablets printed with 90% infill, 80% isoniazid release was observed between 40 and 852 min. Drug release characteristics could further be altered by changing the infill or the size of the printed tablets allowing personalization of the tablets. This study presents novel formulations containing isoniazid for prevention of latent tuberculosis and investigates 3D printing technology for personalized production of oral solid dosage forms enabling adjustable dose and drug release properties.

**Electronic supplementary material:**

The online version of this article (10.1208/s12249-018-1233-7) contains supplementary material, which is available to authorized users.

## INTRODUCTION

Tuberculosis, an infectious disease caused by *Mycobacterium tuberculosis* typically affecting the lungs, is the ninth most common cause of death worldwide (1). According to estimations by the World Health Organization (WHO), 10.4 million people fell ill with tuberculosis in 2016 and almost two million deaths were estimated among the people infected with the disease. Most deaths caused by tuberculosis could successfully be prevented with early diagnosis, suitable treatment, and prevention of latent tuberculosis in identified risk groups. Therefore, preventive treatment for tuberculosis and latent tuberculosis is needed (1). Treatment of latent tuberculosis with isoniazid monotherapy daily for 6 months is recommended for both adults and children in countries with both high and low disease prevalence. Currently, the recommended isoniazid dose for latent tuberculosis is 5 mg/kg for adults and 7–15 mg/kg for children with a maximum dose of 300 mg/day (2).

Age has been reported to have an effect on the pharmacokinetics of isoniazid since the drug is metabolized more rapidly in children (3) due to, *e.g.*, increased first pass effect as children have a larger liver in comparison to their body weight as compared to adults (4). Other factors that have been identified to affect the isoniazid dose required are the maturation of the N-acetyl transferase 2 (NAT2) enzyme, the acetylation rate (fast, intermediate, and slow) of isoniazid due to different genotypes of NAT2, and body surface area to name a few (3–5). Isoniazid is listed on the Best Pharmaceuticals for Children Act, which is a priority list of needs in pediatric therapeutics for 2017 given out by the National Institutes of Health, where there has been identified a scientific need to advance the technology and design of the dosage forms to improve adherence and effectiveness of the isoniazid therapy (6). Individualized doses are needed in the treatment of tuberculosis as it has been reported that fast acetylators of isoniazid typically show subtherapeutic plasma exposure values where on the other hand, slow or intermediate acetylators may require a lower dose to achieve the recommended plasma values of 3–6 mg/L (7). Individualized drug therapies may help to solve the recognized unmet need for isoniazid as personalized therapies not only increase the compliance but also the effectiveness and success of a therapy (8,9). It has moreover been reported that over 80% of the adverse effects may originate from inappropriate dosing or dose combinations (10). These factors highlight that personalized treatment of isoniazid may lead to better treatment outcomes, especially in children who most likely will undergo physiological changes during the time span of the lengthy treatment. Not only the dose but also the tablet geometry can be adjusted during the treatment regimen to tailor the drug release if so required for the treated patient.

Three-dimensional (3D) printing, a rapid prototyping technology based on principles of additive manufacturing that allows the manufacturing of dosage forms with different geometrical shapes, has recently been explored in the biomedical and pharmaceutical field (8,11–15). 3D printing is attractive to scientists and manufacturers in the pharmaceutical field that currently are bound to conventional manufacturing methods that produce fixed-dose tablets with limited possibilities of personalization (16–20). Manufacturing of small batches or even a single dose utilizing 3D printing is considered cost-effective (13,21,22) compared to traditional pharmaceutical manufacturing methods that on the contrary are cost-effective when large batches of the same dosage form (same size and dose level) are produced. Production of small batches plays a vital role in personalized drug therapies, which need to be produced on-demand and close to the patient to be able to prepare patient-tailored treatments needed at that specific time (16,17).

Initially, 3D printing in the pharmaceutical field mainly involved printing of drug-loaded commercially available filaments that were developed for the fused deposition modeling (FDM) 3D printers (*e.g.*, polylactic acid, acrylonitrile butadiene styrene, and polycaprolactone) (18,23,24). These types of polymers typically displayed a sustained drug release that is more suitable for implants than oral dosage forms. The oral route for administration of drugs still today remains the favored choice by patients (19,25). A logical trend is, therefore, to move towards studying polymers that would be suitable for 3D printing of oral dosage forms as well as utilizing methods that allow for sufficient drug loading compared to the soaking methods previously used to incorporate the drug in the commercially available filaments**.** Lately, various polymer–drug combinations have been hot-melt extruded (HME) and their suitability for FDM printing of oral tablets has been explored (26–32). Readers interested in additional applications of pharmaceutical 3D printing are referred to the following extensive reviews (21,22,33,34).

The aim of the current study was to produce FDM 3D printable filaments consisting of FDA-approved pharmaceutical grade polymers by utilizing hot-melt extrusion. The goal was to formulate and produce filaments with sufficient drug loading and variable release characteristics (immediate and controlled release) that subsequently could be used in a 3D printer to produce differently sized solid oral dosage forms with a relevant dose of isoniazid. Furthermore, the effect on the release properties originating from the printing process itself at elevated temperatures as well as different properties of the printed tablets, namely infill and tablet size, was studied. In addition, the printability with regard to the mechanical properties of the produced filaments was investigated. To the best of our knowledge, there are no previous studies on 3D-printed oral isoniazid dosage forms that could have potential in manufacturing at hospital pharmacies, wards at the point-of-care, or which could contribute to on-demand dose dispensing in developing countries.

## MATERIALS

Isoniazid > 98% was purchased from TCI America (Portland, OR, USA). Hydroxypropylmethylcellulose (HPMC, Benecel™ grade E5 Pharm and K100M Pharm) and hydroxypropylcellulose (HPC, Klucel™ grade EF Pharm, MW 80,000 and HF Pharm, MW 1,150,000) were donated by Ashland (Covington, KY, USA). Polyethylene oxide (PEO, Sentry™ Polyox™ WSR N-80 NF, MW approx. 200,000, and Sentry™ Polyox™ WSR N-750 NF, MW approx. 300,000) was supplied by Dow Chemical Company (Midland, MI, USA). Eudragit® RS PO, RL PO and L 100 were kindly provided by Evonik Industries AG (Essen, Germany). Triethyl citrate ≥ 99% (TEC) was purchased from Sigma-Aldrich (Darmstadt, Germany), and Kolliphor® TPGS (vitamin E polyethylene glycol succinate, d-alpha tocopherol content min 250 mg/g) was acquired from BASF (Ludwigshafen, Germany). Buffering agents, potassium dihydrogen phosphate and sodium hydroxide pellets were of analytical grade and purchased from Merck (Darmstadt, Germany) and Sigma-Aldrich (Darmstadt Germany), respectively. Polylactic acid (PLA) 3D printable filament (PLA natural) with a diameter of 1.75 mm obtained from MakerBot (MakerBot Industries, NY, USA) was used as received.

## METHODS

### Hot-Melt Extrusion

A total of 13 formulations containing 30% (*w*/*w*) isoniazid and varying polymers and plasticizers were prepared in 50 g batches (Table I). The powder blends were mixed in a closed plastic container on a Maxiblend (GlobePharma, New Brunswick, NJ, USA) at 25 rpm for a minimum of 20 min before being extruded as cylindrical filaments utilizing a Thermo Fisher Process 11 mm co-rotating twin-screw extruder (Waltham, MA, USA). A standard screw assembly with three mixing zones was used. Barrel temperatures between 100 and 155°C and a screw speed of 50 rpm were used to extrude the different formulations. Polymer melts were extruded through a circular die (⌀ 2 mm) and guided onto a conveyor belt for cooling and fine-tuning of the filament diameter. The diameter of the extruded filament was adjusted by changing the speed of the conveyor belt. The hot-melt extruded filaments were coiled, stored in sealed zip lock bags protected from light, and subsequently used as feedstock material for the FDM 3D printing.

**Table I:** Process Temperatures and Formulation Compositions for the Hot-Melt Extruded Formulations as well as Subsequently Applied Temperatures During the Printing Step

Formulation	Drug	Polymer(s)	Plasticizer	HME T (°C)	Print T (°C)
1	30%	50% HPC EF + 20% HPMC E5		150	185
2	30%	70% PEO N80		140	185
3	30%	65% HPC EF + 5% PEO N80		140	190
4	30%	60% HPMC E5 + 10% PEO N80		140	–
5	30%	40% HPC EF + 30% PEO N80		140	195
6	30%	40% HPMC K100 + 20% PEO N80	10% TPGS	120	–
7	30%	40% HPMC K100 + 30% PEO N750		155	–
8	30%	45% HPC HF + 25% PEO N750		130	185*
9	30%	20% RS PO + 20% RL PO +30% PEO N750		140	–
10	30%	60% HPC EF + 10% PEO N80		130	185
11	30%	20% RS PO + 20% RL PO +27.5% PEO N750	2.5% TEC	100	165
12	30%	40% HPC HF + 30% L100		130	170
13	30%	40% HPC HF + 30% E PO		130	175

*HME* hot-melt extrusion, *T* temperature

*Formulation 8 showed day-to-day variability regarding the printability and should therefore be considered as non-printable

### Three-Point Bend Test

Cylindrical extruded filaments were tested regarding their mechanical properties utilizing a three-point bend test with a TA-XT2i texture analyzer (Texture Technologies, Hamilton, MA, USA). Approximately 45-mm-long filament pieces were cut from the extruded filament strand and placed on the three-bend rig sample holder (TA-95N, Texture technologies) with a set gap of 25 mm. The measurements were initiated when the trigger force of 5.0 g was exceeded and were conducted with a speed of 10 mm/s. The measurement endpoint was set to 15 mm. Measurements were repeated ten times for each formulation, and the Exponent software (version 6.1.5.0, Stable Micro Systems, Godalming, UK) was used for data analysis.

### Vapor Sorption Analysis

To evaluate the moisture uptake of the prepared filaments, dynamic vapor sorption analysis was performed using SPS11 (ProUmid, Ulm, Germany). For analysis, samples were dried to 0% relative humidity (RH). Afterwards, RH was automatically increased up to 90% in steps of 10% RH, when equilibrium (0.0100%/30 min) was reached for the set climate stage. The time between weighing cycles was 10 min, and the minimum and maximum time per climate cycle were 60 min and 72 h, respectively.

### 3D Printing

A MakerBot Replicator 2 (MakerBot Industries, Brooklyn, NY, USA) with custom-built air-cooled print head was used for 3D printing of filaments replicating the feedstock filament and various sized tablets. Prior to the printing step, the 3D models were designed in Rhinoceros (version 4.0). The designed replicated filaments were 40 mm long and 1.75 mm in diameter, while tablets were designed to be either 6, 8, or 10 mm in diameter and 2.5 mm in height. The designs were saved as stereolithography (.stl) files and exported into the MakerBot Desktop software (version 3.7.0.108) where printing parameters were determined. The printing temperature was varied depending on the formulation from 165 to 195°C (Table I), the set layer height was 0.05 mm, the number of outlines printed on each layer was set to two, and printing and traveling speed of the print head was 90 mm/s and 150 mm/s, respectively. Two different infill levels were investigated, 15% and 90%, to achieve tablets with different porosities**,** as the infill level is an indicator of how densely the material is being deposited inside the printed outlines. The infill level for the 3D-printed filament strand was set to 90% to mimic the dense structure of the hot-melt extruded filament. Filaments and tablets were printed on a build platform of glass covered with blue tape (MakerBot Industries) to improve the adhering to the build platform.

### Differential Scanning Calorimetry

Differential scanning calorimetry (DSC) was performed to evaluate thermal properties of the samples (raw materials, hot-melt extruded filaments as well as 3D-printed tablets) using the DSC 1 (Mettler Toledo, Columbus, OH, USA). Samples (3 to 6 mg) were analyzed in pierced aluminum pans in a heat-cool-heat cycle from − 20 to 250°C with a heating and cooling rate of 10°C/min and 20°C/min, respectively. The obtained data was further analyzed in the STARe software (Version 9.20, Mettler Toledo).

### Drug Content

Drug content of the feedstock material used for 3D printing was measured in distilled water in order to evaluate the uniformity of the prepared hot-melt extruded filaments. Briefly, 150 ± 6 mg of hot-melt extruded filaments pieces, taken from random sections of the extruded strand, were placed in 100 mL of distilled water and shaken at 120 rpm (Multi-shaker PSU 20, Biosan, Latvia) for a minimum of 24 h. Samples were diluted and subsequently measured utilizing a UV-Vis spectrophotometer (Lambda 35, PerkinElmer, Singapore) at 263 nm. Ten replicates of each formulation were analyzed.

Drug content data for the 3D-printed tablets was obtained from the dissolution study. Concisely, once the dissolution for each tablet had reached a plateau around 100% drug release, the mean value of five subsequent time points was calculated and reported as the drug content (mean ± SD, *n* = 3). Since the drug release for formulation, 13 had not reached 100% for all the tablets after 24 h; the tablets were kept in the dissolution vessels for another 22 h in room temperature, and the absorbance was then measured manually.

### *In Vitro* Drug Release

*In vitro* drug release studies were performed for the pure drug as received, hot-melt extruded filaments, 3D-printed filaments, as well as 3D-printed tablets to assess the drug release profiles from the formulations. Samples were accurately weighed and placed in the vessels containing 900 mL of phosphate buffer (pH 7.4) at 37 ± 0.5°C with paddles rotating at 100 rpm (Sotax AT 7smart, Basel, Switzerland). Spiral capsule sinkers were used for the 3D-printed tablets while the pure drug and prepared filaments were measured as such. Samples of the release media were automatically withdrawn with a pump (Sotax CY 6, Basel, Switzerland), filtered (glass microfiber filter GF/B, GE Healthcare Life Sciences, UK), and measured at predefined time points utilizing an online UV-Vis spectrophotometer (Lambda 35, PerkinElmer, Singapore) at 263 nm. Samples were measured in triplicate. The percent of drug released at the specific time points was calculated based on the theoretical drug content (30% *w*/*w*) in the samples.

## RESULTS AND DISCUSSION

### Hot-Melt Extrusion and 3D Printing

The FDM 3D printing process uses filament strands as feedstock material. Hot-melt extrusion, which is an established manufacturing method in the pharmaceutical industry, was used to homogeneously incorporate the drug in a polymer matrix and simultaneously produce filament strands that could be used in the printing process. In this study, 13 formulations consisting of pharmaceutical grade polymers were successfully hot-melt extruded with a drug loading of 30% (*w*/*w*). One drug loading was used to support the basic scenario that this kind of manufacturing technique would be used in a hospital pharmacy setting where fixed-dose filaments would be provided for 3D printing of flexible dose tablets. An increased or decreased drug loading would alter the drug release as previously reported (18,35). A 30% (*w*/*w*) drug load was expected to be sufficient to achieve an adequate amount of drug in the final dosage form, although still resulting in drug release differences between the formulations originating from the polymers used.

The desired filament diameter was 1.75 mm for optimal performance in the MakerBot 3D printer as stated by the manufacturer. Polymer properties, *e.g.*, die swell observed for viscoelastic materials, will have an effect on the diameter of the extruded filament. As the screw speed and die size used in this study were the same for all formulations, the speed of the conveyor belt was adjusted to obtain filaments within the desired diameter range. It was observed that if the filament diameter was too small, the output of material from the printer was decreased and too large of a filament diameter led to no extrusion of material, which in both cases caused fluctuation in material flow and typically failed printings. An uneven filament diameter has previously been reported to cause inconsistent deposition of the material and therefore, poor printing outcome as the filament diameter will determine the feeding rate of the filament (36). The diameter of the produced filaments is consequently of high importance to achieve accurate and successful printing, which naturally is required in the pharmaceutical industry.

**Fig. 1:**
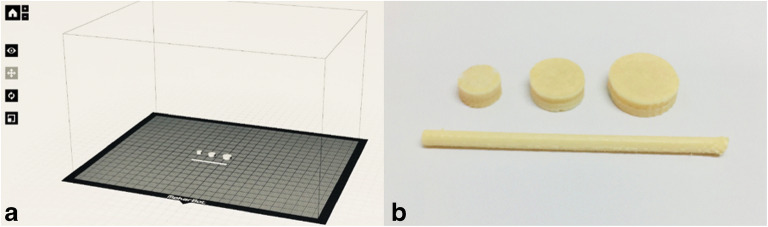
**a** Three different sized tablets as well as a filament strand were designed and imported into the printing software where the printing parameters were determined. **b** The designs were subsequently printed with drug-loaded filaments and further analyzed

Three different tablet sizes (⌀ 6, 8, and 10 mm), each with two different infill levels (15% and 90%) were printed to support the scenario that hospital pharmacies could 3D print tablets of different doses and properties using pre-made filaments. Additionally, cylindrical strands representing the hot-melt extruded filaments were printed (Fig. 1) in order to get an understanding of whether the 3D printing process, at elevated temperatures, would affect the release properties when the geometry and therefore the surface area was kept unchanged. An increased temperature was required to efficaciously 3D print the filaments compared to the temperature required for hot-melt extrusion of filaments, which can be explained by the lack of shear stress during printing as compared to the hot-melt extrusion process (Table I).

Eight out of the 13 extruded formulations were successfully printed into the described geometries. The weight of the different sized tablets varied depending on the formulation and the printed infill from 59.2 ± 0.5 mg to 88.7 ± 2.7 mg, 107.6 ± 1.3 mg to 145.2 ± 1.0 mg, and 143.8 ± 1.2 mg to 236.7 ± 3.1 for the ⌀ 6, 8, and 10 mm tablets, respectively (Table II). The weight differences between the printed tablets for the different formulations most likely originate from the different melt flow properties of the prepared filaments at the applied temperatures. Melt flow behavior of a material, that is temperature dependent, has been identified as an important parameter in 3D printing affecting the printability and quality of the printed object (36,37). It is worth noticing that all the different formulations in this study, with an exception of the printing temperature, were printed with the same settings (settings optimized for PLA by the manufacturer) and that rheological studies of the produced filaments preferably should be carried out to successfully optimize the printing parameters for the different formulations to further improve the printing outcome. However, when three different sized tablets were printed for each formulation, a linear increase in the tablet weight was observed, indicating that aimed doses of isoniazid can be fabricated utilizing FDM 3D printing. Moreover, *R*^2^ values for the printed formulations were for most formulations in a similar range as the reference material PLA underlining that the prepared filaments were as suitable for FDM 3D printing as the optimized material with regard to manufacturing of tablets with different doses. Tablets containing isoniazid were printed in batches of three tablets per batch. The standard deviation was generally low within the batch, but a more substantial deviation could be seen for some of the formulations between the printed batches (data not shown). The greater deviation between the printed batches may be explained by the fact that the produced filaments were uneven in diameter or due to poor flow of the material in the print head after long printing times. Furthermore, as the printing took place in a non-controlled environment, day-to-day changes in the ambient temperature and humidity were likely to have an effect on how the filament behaved in the printer. Filament optimization and a printer with a controlled environment in the printing chamber would, therefore, be beneficial to further advance the printing outcome.

**Table II:** Weights of the 3D-Printed Tablets for the Different Formulations and Their Size and Weight Relationship, Mean ± SD, *n* = 6 (*n* = 3 for PLA)

	6 mm, 15% (mg)	6 mm, 90% (mg)	8 mm, 15% (mg)	8 mm, 90% (mg)	10 mm, 15% (mg)	10 mm, 90% (mg)	*R*^2^, 15%	*R*^2^, 90%
PLA	76.4 ± 0.6	83.9 ± 0.5	135.8 ± 0.2	150.1 ± 1.1	210.3 ± 1.3	235.1 ± 2.7	0.9958	0.9948
1	88.7 ± 2.7	77.7 ± 3.4	141.2 ± 4.5	145.2 ± 1.0	181.5 ± 2.2	220.8 ± 4.5	0.9942	0.9989
2	61.6 ± 0.7	65.3 ± 0.6	107.6 ± 1.3	116.2 ± 5.3	143.8 ± 1.2	170.0 ± 0.8	0.9952	0.9997
3	59.2 ± 0.5	69.4 ± 5.3	133.2 ± 0.4	141.4 ± 2.0	195.0 ± 7.2	192.0 ± 8.1	0.9973	0.9900
5	59.2 ± 1.7	67.8 ± 2.2	140.0 ± 3.4	144.5 ± 4.1	203.6 ± 12.6	236.7 ± 3.1	0.9996	0.9972
10	70.8 ± 1.0	73.5 ± 0.9	121.5 ± 2.4	130.1 ± 2.1	193.4 ± 5.2	205.9 ± 1.7	0.9902	0.9930
11	66.1 ± 1.2	73.7 ± 2.1	141.4 ± 4.9	136.5 ± 0.6	208.3 ± 10.0	222.5 ± 1.9	0.9988	0.9920
12	68.1 ± 2.6	74.7 ± 0.7	115.9 ± 1.8	133.4 ± 4.8	196.5 ± 8.1	214.8 ± 4.7	0.9786	0.9913
13	64.5 ± 0.7	73.6 ± 2.6	130.0 ± 3.7	143.4 ± 5.4	163.1 ± 2.1	165.7 ± 0.9	0.9651	0.9182

### Printability

Formulations composed of HPC showed good printability as six out of the eight printable filaments in this study contained HPC. These formulations contained 40–65% (*w*/*w*) HPC (EF or HF), and it is thus evident that both of these HPC grades possesses good material properties for 3D printing. HPC is a non-ionic water-soluble cellulose ether with versatile properties (38). It has glass transition temperatures at 0°C (originating from a beta transition) and 120°C which make it easily extrudable as the viscosity of the melt significantly drops at the applied temperatures during printing. Furthermore, the beta transition around 0°C results in increased flexibility of the polymer, which is desired for successful FDM 3D printing. Formulations containing HPMC were not printable, except for formulation 1, containing only 20% (*w*/*w*) HPMC and a greater portion of HPC (50% (*w*/*w*)). Hence, HPMC was found to be an unsuitable polymer for printing applications in the range of 40–60% (*w*/*w*) in this study. The addition of 10% (*w*/*w*) plasticizer to formulation 6 resulted in a filament that was too soft. Previous studies have reported successful 3D printing of HPMC (28,39–41). However, the composition of the formulations reported was different than the ones used in this study. Formulations containing PEO in the range of 5–70% (*w*/*w*) were identified as printable when PEO was used as a single polymer together with the drug or in combination with HPC. Formulations containing PEO and HPMC were not printable, which may be attributed to a high concentration of HPMC in the formulations (40–60% (*w*/*w*)) as compared to PEO (10–30% (*w*/*w*)). Formulation 9 containing PEO (30% (*w*/*w*)), Eudragit RS PO (20% (*w*/*w*)), and RL PO (20% (*w*/*w*)) was not successfully printed but could be printed when 2.5% (*w*/*w*) of the plasticizer TEC was added to the formulation prior to extrusion. Other Eudragit grades were also successfully printed when combined with HPC HF.

The printability of a material in a FDM 3D printer is dependent on many factors. Fuenmayor *et al*. (36) suggested that the material should be considered in relation to the feeding, heating, and deposition processes during 3D printing as all of these bring specific challenges. A printable filament should have properties suitable for all of the abovementioned process zones. Aho *et al*. (42) have proposed that rheological studies in combination with thermal and mechanical studies can be used to predict the extrudability and 3D printability of formulations. In this study, to gain initial understanding about the printability of produced filaments, their mechanical properties as well as tendency to absorb or adsorb moisture were studied.

### Mechanical Testing of the Prepared Filaments

To successfully be able to load and subsequently 3D print the prepared formulations, the filament needs be flexible but not too soft (43). A brittle filament will break under the pressure from the driving gears and prevent feeding, while a too soft filament may inhibit feeding when the material is squeezed between the driving gears (28,36). In this study, a three-point bend test setup was used as a fast method to get an initial understanding of the mechanical properties of the prepared filaments. Commercially available PLA filaments that possess optimal mechanical properties for the used printer were chosen as a reference material. When comparing the printability of the different formulations with the breaking distance values gained from the three-point bend test, it was observed that filaments with a higher breaking distance (toughness) had a tendency to be printable while filaments with a breaking distance below 1.5 mm were too brittle to be loaded into the print head of the 3D printer (Table III). This is in accordance with what has been reported by Zhang *et al*. (28) for another type of FDM 3D printer where it was found that the produced filaments should be sufficiently stiff possessing a breaking stress greater than 2941 g/mm^2^ and a breaking distance over 1 mm for successful loading and printing. Another study reported that filaments should have a breaking distance over 1.125 mm to ensure printability (44). The obtained values for breaking stress, representing the stiffness of the filaments were on the other hand not in accordance with the findings reported by Zhang *et al*. (28). The breaking stress for the printable filaments in this study was in the range of 3126–7638 g/mm^2^, while the filaments that were not successfully printed had breaking stress values between 1335 and 5119 g/mm^2^. As these results show no clear trend, it can be concluded that the printability of the filaments is a complex matter where more than one mechanical parameter needs to be taken into account to be fully understood. It was detected that even though a filament may have a sufficient breaking distance, it may be too soft for successful printing leading to deformation of the filament under the pressure of the driving gears. This will result in unsuccessful feeding of the filament, as it can no longer be gripped by the driving gears. Some filaments were also observed to be subjected to wear deriving from the driving gears, seen as small filament pieces being worn off the surface of the filament, which also eventually resulted in failed feeding of the filament towards the nozzle. Based on this study, the fast three-point bend test can be used as an initial indicator regarding printability; however, as the breaking distance is a measure of brittleness, additional mechanical tests concerning the material properties such as the column strength, Young’s modulus, and softness of the filaments should also ideally be performed (36,44).

**Table III:** The Brittleness and Stiffness of the Prepared Hot-Melt Extruded Filaments Were Determined by a Three-Point Bend Test Where the Distance (mm) Until the Filament Broke and Breaking Stress (g/mm^2^) Was Recorded and Used as an Indicator if the Filament Would Be 3D Printable (Mean ± SD, *n* = 10)

Formulation	Breaking distance (mm)	Breaking stress (g/mm^2^)	Printable
PLA	3.69 ± 0.28	15,361.45 ± 2064.55	Yes
1	2.39 ± 0.43	5456.12 ± 769.68	Yes
2	1.93 ± 0.13	3125.75 ± 185.30	Yes
3	3.50 ± 1.09	5046.78 ± 1324.55	Yes
4	0.83 ± 0.05	3145.73 ± 265.98	No
5	2.80 ± 0.62	4982.93 ± 604.85	Yes
6	0.90 ± 0.19	1334.48 ± 67.08	No
7	2.70 ± 0.47	5119.04 ± 369.75	No
8	4.07 ± 0.94	4662.66 ± 625.93	No*
9	1.24 ± 0.13	4251.27 ± 929.07	No
10	2.59 ± 0.32	3255.59 ± 294.42	Yes
11	2.31 ± 0.31	4988.52 ± 142.70	Yes
12	1.78 ± 0.26	7637.32 ± 808.91	Yes
13	2.93 ± 0.28	6496.31 ± 443.79	Yes

*Day-to-day variability regarding printability, as the filament deformed during feeding, which occasionally failed the prints

### Vapor Sorption

**Fig. 2:**
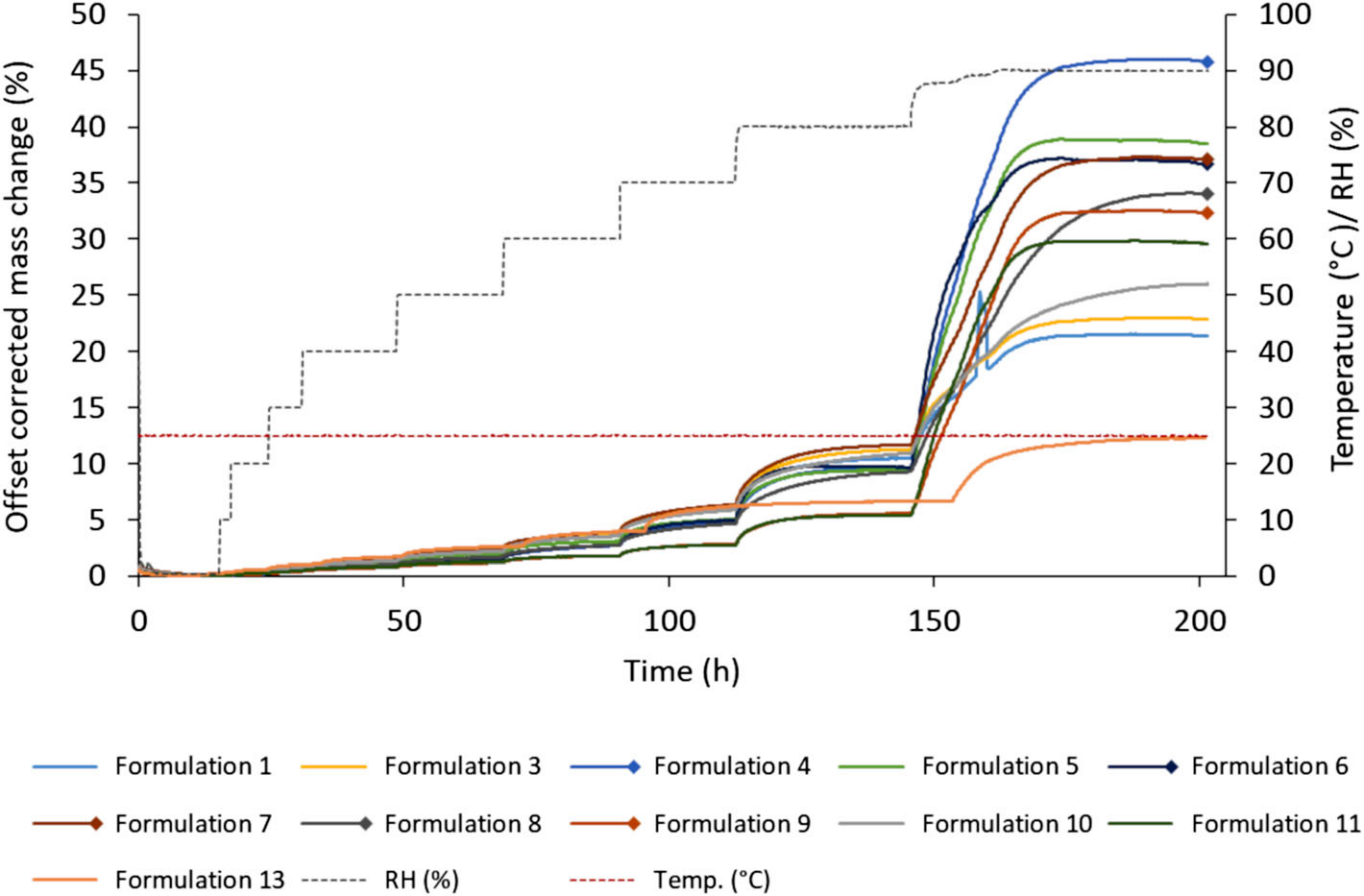
Mass change (%) with offset correction over time (h) at different RHs for the hot-melt extruded filaments. Formulations 2 and 12 are missing from the graph, due to technical problems during the measurement. However, data regarding mass change *vs* RH for these formulations can be found in the supplementary material. Unprintable formulations are marked with a square at the end of the line. *RH* relative humidity

Water uptake for solid dosage forms is of interest as it may negatively affect the physicochemical, chemical, and microbiological stability of the product (45). As new manufacturing methods are introduced to the pharmaceutical field, new process challenges are also encountered. For FDM 3D printing, poor quality, failed prints, and reduced printing times have been reported due to possible moisture uptake of the feedstock material as some polymers are known to readily absorb moisture from the air (46). The amount of moisture absorbed is dependent on multiple factors such as relative humidity, temperature, affinity between the surface of the material, and the water molecules as well as the surface area of the sample (47). Absorbed moisture has been reported to cause a slight increase in the filament diameter as well as fluctuations in the glass transition temperature for the filament leading to inconsistent printing performance (48). Consequently, day-to-day variations regarding printability and quality are to be expected for some materials if the printing process does not take place in a controlled environment. In this study, the hot-melt extruded filaments were analyzed with regard to their moisture uptake to investigate if it could be linked to the printability of the filaments. All prepared filaments absorbed moisture in a sigmoidal manner with low moisture uptake at low humidity and high uptake at higher RH (Fig. 2), which is typical for cellulose- and starch-based polymers (45). At low RH, the increase in mass (%) for the different formulations was small, *e.g.*, between 0.33% and 0.71% at 30% RH and even at 60% RH, the change of mass was less than 4% for all of the formulations (data for all RH studied available in supplemental 1). At 90% RH, however, the moisture uptake was rapidly intensified for all the filaments. Formulation 4 displayed the greatest weight increase at 90% RH with a total increase of 45.78%. No clear trend could be seen regarding printability and moisture uptake at low RHs. However, based on the data obtained from this study, it appears that filaments that have a high total uptake of moisture have a tendency to be unprintable. A common factor for all the formulations with a high moisture uptake at high RH was that they all contained either PEO N750 or HPMC E5 or K100, indicating that the major moisture uptake can be attributed to these polymers.

### DSC

**Fig. 3:**
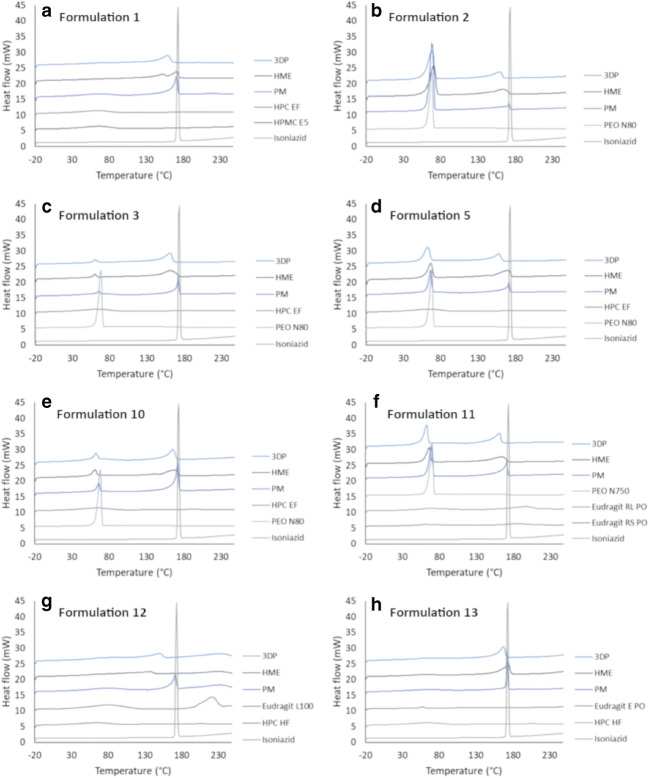
Thermograms (endo up) from the first heating run for the different printable formulations (**a**–**h**). Raw materials are presented in grey and formulation mixtures in blue. *PM* physical mixture, *HME* hot-melt extruded filament, *3DP* 3D-printed tablet

The thermal properties of the raw materials, hot-melt extruded filaments, as well as 3D-printed tablets were investigated in order to gain information about how the formulation changed during the different processing steps. Unprocessed isoniazid melted during the first heating run with a sharp endothermic peak onset at 170.5°C and a max peak located at 170.7°C (Fig. 3). The DSC thermograms from the first heat scan were similar for the hot-melt extruded filaments as when they were further processed into 3D-printed tablets. This indicates that no major changes take place when the material is reheated to elevated temperatures during 3D printing of the material, it would appear that no degradation of the drug occurs during 3D printing. From the thermograms of the physical mixtures, hot-melt extruded filaments as well as the 3D-printed tablets, it is evident that the drug starts dissolving in the polymer around 100°C before finally reaching a larger endothermic peak identified as the melting point of isoniazid. Generally, a small melt point depression of the drug as well as broadening of the endothermic peak for the hot-melt extruded filament as compared to the physical mixture was observed. Further melt point depression was discovered for some of the 3D-printed tablets compared to the hot-melt extruded filament, when being further processed at temperatures close to or above the melting point of the drug. This can be attributed to an increased amount of the drug being dissolved or dispersed in the polymer matrix resulting in a reduction of the chemical potential of the system due to interaction between the materials. Other endothermic events present during the first heating were typically broad and located between 15 and 100°C, which originate from dehydration of the polymers due to the hygroscopic nature of the polymers present in the formulation (29). Other, more sharp, melting peaks could be attributed to melting of the polymer as they were in agreement with the melting peak present for the pure polymer. No completely amorphous formulations were obtained, neither by hot-melt extrusion nor by further 3D printing the material as all formulations showed endothermic peaks during the first heating. However, as isoniazid is a highly water-soluble drug, the main goal of utilizing hot-melt extrusion in this study was to prepare drug-loaded filaments suitable for 3D printing rather than create amorphous formulations.

During the second heating of the material, the melting peaks were in general smaller than during the first run and also amorphous material was formed as no melting or crystallization peaks were present on the thermograms (supplemental 2). The single phase formed during the second heating when the thermal history of the material was removed could either be seen for all types of formulations (physical mixture, hot-melt extruded filament, or the 3D-printed tablet) or only for the hot-melt extruded filament and/or 3D-printed tablets that were processed at high temperatures.

The only formulation where a distinctive change (except for melt point depression) between the hot-melt extruded filament and the 3D-printed tablet was observed during the first heating was formulation 1 containing isoniazid, HPC EF, and HPMC E5. The thermogram for the 3D-printed tablet showed that the drug started melting in the polymer at around 70°C. The melting peak onset and the max peak are located at 139.4°C and 159.1°C, respectively. The hot-melt extruded filament of formulation 1, on the other hand, displayed a split melting peak with max peaks located at 159.1°C and 171.3°C, respectively. The difference between the thermograms for the hot-melt extruded filament and the 3D-printed tablet may be explained by the higher temperature applied during 3D printing compared to the hot-melt extrusion process. However, since the physical mixture of formulation 1 did not possess a split peak, but one single melting peak, it is more likely that a phase separation between the polymer and the drug had occurred during long storage times of the filament and that a single phase was reformed during the printing process when high temperatures were applied.

### Drug Content of Hot-Melt Extruded Filaments and 3D-Printed Tablets

All produced hot-melt extruded filaments and 3D-printed tablets had a target drug load of 30% (*w*/*w*) isoniazid in order to obtain a sufficient amount of drug in the final tablets. The produced filaments that were printable and the 3D-printed tablets were confirmed to have a drug load between 27.9 ± 2.6% and 31.7 ± 0.2% and 27.5 ± 1.1% and 34.7 ± 0.2%, respectively (Table IV). Hot-melt extruded filament strands and subsequently 3D-printed tablets displayed a drug load in the same range, suggesting that no drug degraded during the printing process. Overall, the 3D-printed tablets had a smaller standard deviation than the starting material, which may be explained by the fact that for printing, a smaller length of the filament was used as compared to the filament content analysis where the filament pieces at random locations were cut off from the filament strand. However, the generally small standard deviation indicated that the hot-melt extrusion process produced homogenous filaments.

**Table IV:** Drug Content (%) for 3D-Printed Tablets (Different Sizes and Infill Levels) as well as Hot-Melt Extruded Filaments That Served as Starting Material in the Printing Process

	% API in 3D-printed tablets and hot-melt extruded filament (*w*/*w*)						
	6 mm, 15%	6 mm, 90%	8 mm, 15%	8 mm, 90%	10 mm, 15%	10 mm, 90%	Filament
1	31.6 ± 0.2	28.0 ± 1.7	30.4 ± 0.2	31.2 ± 1.4	27.5 ± 1.1	29.9 ± 1.3	27.9 ± 2.6
2	30.6 ± 0.3	30.8 ± 0.1	30.1 ± 0.7	30.9 ± 0.0	30.4 ± 0.1	30.6 ± 0.1	30.2 ± 1.0
3	31.0 ± 0.1	30.9 ± 0.1	31.1 ± 0.1	31.0 ± 0.0	30.9 ± 0.1	30.7 ± 0.1	31.7 ± 0.2
5	30.3 ± 0.5	30.5 ± 0.1	30.6 ± 0.2	30.8 ± 0.0	31.3 ± 0.0	30.7 ± 0.1	29.6 ± 1.2
10	34.7 ± 0.2	34.6 ± 0.1	32.8 ± 0.4	33.4 ± 0.4	34.3 ± 0.1	30.4 ± 0.4	31.3 ± 1.8
11	31.8 ± 0.1	32.1 ± 0.1	31.6 ± 0.0	30.9 ± 0.0	31.2 ± 0.1	31.0 ± 0.1	31.0 ± 0.3
12	30.2 ± 0.4	30.9 ± 0.1	31.1 ± 0.1	30.3 ± 0.0	30.4 ± 0.1	30.7 ± 0.1	29.7 ± 1.1
13	31.1 ± 0.1	30.8 ± 0.3	30.8 ± 0.1	30.8 ± 0.2	30.9 ± 0.0	30.3 ± 0.1	30.2 ± 0.6

The theoretical drug content for all formulations was 30% (*w*/*w*). Data presented as mean ± SD, *n* = 3 for the 3D-printed tablets and *n* = 10 for the HME filaments

### *In Vitro* Drug Release

**Fig. 4:**
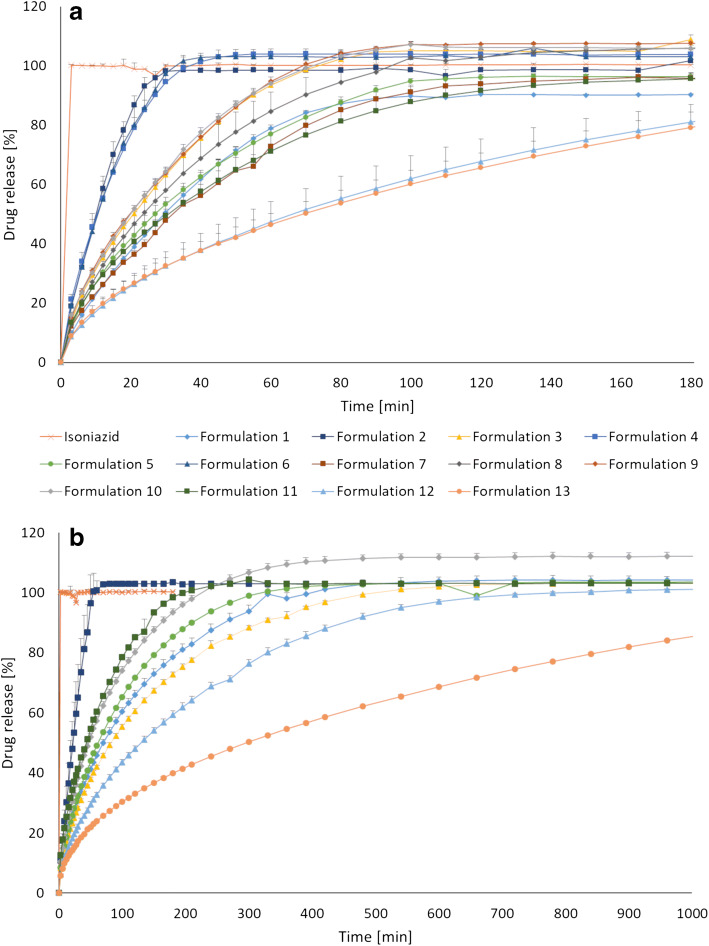
**a** Drug release of isoniazid from hot-melt extruded filaments and **b** drug release from 3D-printed tablets with a size of ⌀ 8 mm and a 90% infill level compared to pure drug. Data presented as mean ± SD, *n* = 3

*In vitro* drug release revealed that hot-melt extruded filaments with different release profiles were successfully prepared, which was even more pronounced when the filaments were further processed into 3D-printed dosage forms (Fig. 4). This showed that both the formulation development and the 3D printing parameters are important factors that will have an effect on how the drug is released. The various 3D-printed tablets presented in Fig. 4b (8 mm, 90% infill) showed an 80% drug release that ranged from 40 to 852 min, which confirms that personalized dosage forms with tailored isoniazid release for prevention of latent tuberculosis may be achieved utilizing 3D printing. All formulations started releasing the drug immediately when placed in the dissolution media, as the drug was present also on the surface of the tablet since no coating was applied. Formulation 2, containing PEO, showed the fastest drug release of all formulations. It was expected that this formulation would release the drug rapidly as PEO is a hydrophilic polymer that hydrates quickly to form a gel layer on the surface of the tablet that facilitates the release of the drug. Since PEO is non-ionic polymer, no interaction between drug and polymers is to be expected (49). The most sustained drug release of the prepared 3D-printed tablets was observed for formulation 13, which did not reach a complete 100% drug release during the 24 h sampling period. This formulation could be suitable for a once daily administration approach, which may be beneficial for adherence. The formulation was projected to show a sustained drug release as HPC HF is a hydrophilic, high molecular weight (MW = 1,150,000) polymer that typically is used in the range of 15–35% (*w*/*w*) for manufacturing of controlled release conventional tablets (38). Eudragit E PO was combined with HPC HF due to its swellable and permeable properties at a pH above 5.0. Furthermore, the polymer acts as taste- and odor-masking agents and protects the drug against light and moisture, which may be advantageous for the light-sensitive drug. In addition, the taste-masking properties can be beneficial with regard to adherence to the treatment. Formulation 12, containing Eudragit (L100) and HPC, released 80% of the drug within 334 min (8 mm, 90%) and was thus the second slowest formulation prepared in this study. Formulation 10 and 11 showed similar release of the drug from the 3D-printed 8 mm, 90% tablets and were the formulations that showed the fastest drug release after formulation 2.

**Fig. 5:**
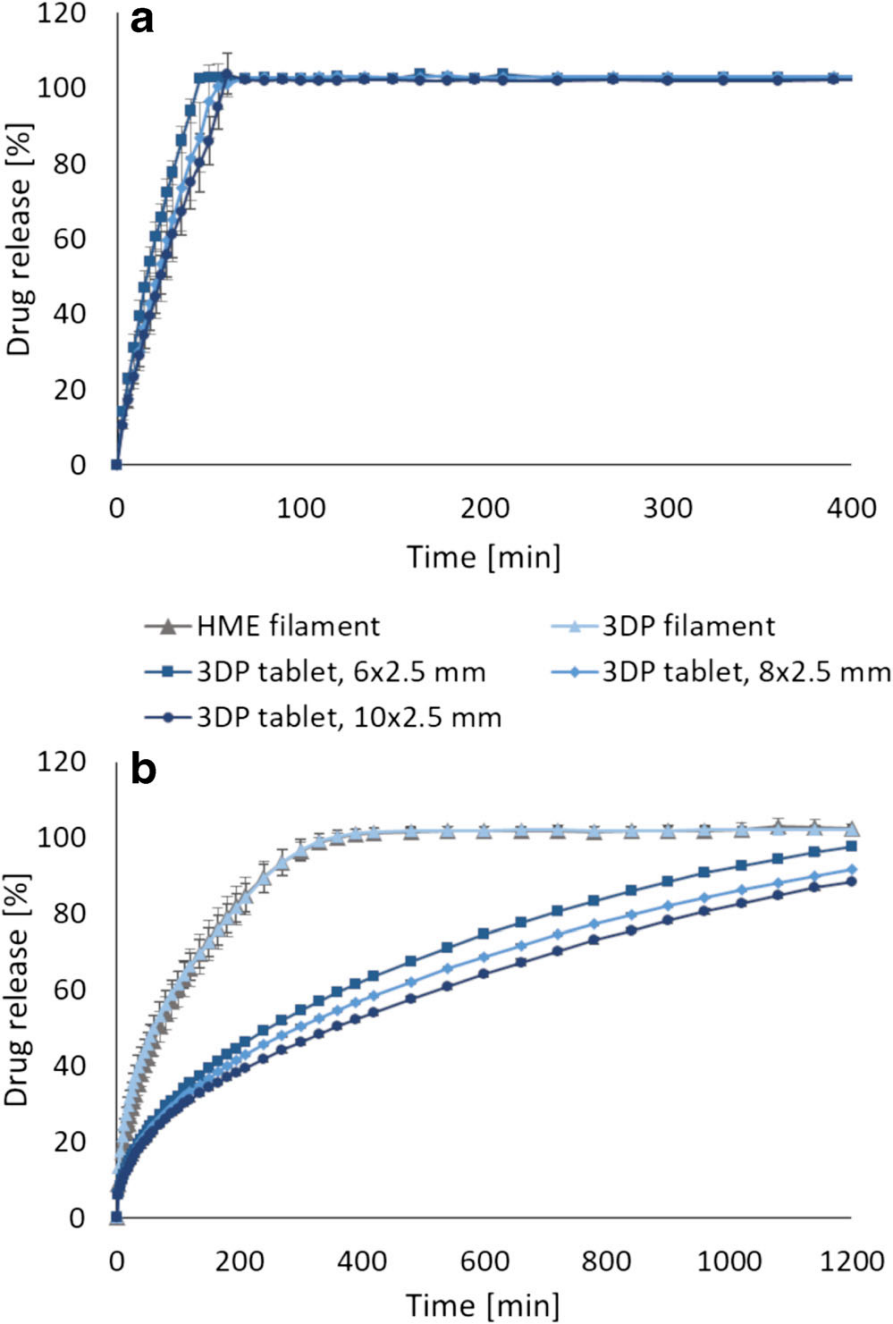
**a** Drug release dependent on tablet size for formulation 2. **b** The difference in drug release for the different sized tablets was more prominent for the sustained release formulation 13. Similar drug release profiles were observed when 3D printing a filament to mimic the original feedstock material. Data presented as mean ± SD, *n* = 3

The impact of the tablet size on the drug release was not very prominent. However, as expected, a trend where ⌀ 6 mm tablets released the drug the fastest and the 10 mm tablet the slowest, which can be attributed to the surface area of the tablets, was observed. It has previously been reported that the geometrical shape of the FDM 3D-printed tablet affects the drug release profile (32,50). The surface area to volume ratio is thus important to take into account when aiming for a certain drug release profile, as an increased surface area to volume ratio have been reported to have a faster drug release. The tablet size and drug release dependency trend was more noticeable for the sustained release formulation than for the formulations that release the drug rapidly as shown in the examples in Fig. 5. The drug release from the hot-melt extruded filaments was in all cases faster than the further processed 3D-printed tablets (Figs. 4 and 5b), which again may be explained by the difference in surface area. When a filament strand was 3D-printed to mimic the filament used as feedstock material, a similar release profile was observed, suggesting that the additional processing of the material at elevated temperatures during the 3D printing step did not alter the drug release properties nor cause degradation of the drug. This is in accordance with the results from the DSC and the drug content analysis. The stability of the drug during 3D printing, despite in some cases exposure to temperatures above the melting point of the drug, may be explained by the short contact time with the heating block during the printing step compared to the hot-melt extrusion process where the material is heated up for a much longer time (30). Moreover, the stability of the drug at higher temperatures may be linked to the lower shear forces present during 3D printing compared to the hot-melt extrusion step.

Printed infill levels have previously been reported to alter the drug release (39,51). In this study, tablets with different inner porosities were printed to understand to which extent the drug release of the printed tablets could be fine-tuned by this feature. Different inner porosities were attained by printing tablets with different infill levels, namely 15% and 90%, resulting in an almost completely void and solid infill, respectively. The drug release studies revealed that the effect of the infill for most formulations was fairly small (supplemental 3), which can be explained by the fact that the default infill setting in the MakerBot Desktop software is to print the set infill level for only 15% of the printed object. It was noticed that during the first 45% of the printed object, the infill level was in fact 100%, and only from 45 to 60% printing occurred with the set infill level (15% or 90%). The rest of the object was then again printed with 100% infill. By increasing the percentage of the tablet that is printed with the set infill level, differences in the release profile of the drug from the printed tablets ought to be greater. It has been reported previously that the difference in drug release was not statistically significant when the tablet was printed with a base and a cap; nevertheless, a much faster drug release was observed when no base or cap, only infill, was printed (39). By printing such tablets, the inner parts of the tablet can immediately come in contact with the dissolution media and be wetted where tablets printed with a solid outer shell will release the drug slower as the surface area in contact with the release media is smaller.

The *in vitro* drug release of the printed dosage forms possessed versatile properties, highlighting the possibility to tailor the dosage form according to the patient’s need and requirements. Depending on the preferences as well as the metabolic rate of isoniazid in the treated patient, one of the presented formulations could be used for prevention of latent tuberculosis as both the dose and drug release can be adjusted. This study presents formulations suitable for a once a day (formulations 12 and 13) as well as multiple times a day (formulations 1, 2, 3, 5, 10, and 11) administering approach. This study furthermore demonstrated that 3D printing enables production of different doses and drug release profiles by simply changing the digital design. The flexibility of 3D printing makes the technique attractive for production of personalized dosage forms compared to conventional manufacturing methods where for example punches and dies need to be changed when a different tablet size is needed.

## CONCLUSIONS

In this study, drug-loaded feedstock material for FDM 3D printing was prepared utilizing hot-melt extrusion. Studying the prepared filaments revealed that breaking distance was a good initial predictor regarding printability and that the moisture uptake of the filaments revealed a trend, where unprintable filaments typically showed a greater total moisture uptake as compared to the printable formulations. Thirteen different formulations with suitable properties for oral drug delivery were produced and eight out of these were subsequently successfully processed into tablets of different sizes and infill levels using a Makerbot 3D printer. All printable formulations showed good correlation between the printed tablet size and mass of the tablet, highlighting the potential to utilize 3D printing for production of personalized doses for prevention of latent tuberculosis. The possibility to easily adjust the dose according to the weight and metabolic rate of the patient is expected to improve the efficacy and adherence to the therapy. In addition, *in vitro* drug release of the printed dosage forms revealed versatile properties, making it possible to administer the dosage form once or multiple times a day depending on the need and requirements of the patient. Due to the sustained isoniazid release, formulations 12 and 13 could be taken once daily, where formulations 1, 2, 3, 5, 10, and 11, that released the drug faster, would be suitable for a multiple times a day administering approach. This study further highlights that by combining formulation development of the feedstock material with the endless geometrical potentials associated with 3D printing, personalized oral dosage forms with nearly limitless properties can be produced.

It has been shown in the present study and previously by other scientists that different release patterns may be obtained by varying the printed structure and infill level. One next step would be to develop a software that could calculate the required mass to be printed as well as the release profile by entering parameters such as feedstock material, dosage form size, infill level, and structure if this treatment decision and manufacturing would be done at the point-of-care. By doing so, 3D printing could become an even more attractive manufacturing method within the pharmaceutical field and could likely take a step towards being an established manufacturing method in, *e.g.*, hospital pharmacies so that patients sooner could benefit from dosage forms tailored according to their needs.

## Electronic Supplementary Material

ESM 1 (DOCX 3477 kb)
