# Supplementary material for: 3D-Printed Isoniazid Tablets for the Treatment and Prevention of Tuberculosis—Personalized Dosing and Drug Release
Source: AAPS PharmSciTech. 2019 Jan 7;20(2):52. doi: 10.1208/s12249-018-1233-7 (PMC6373414; doi:10.1208/s12249-018-1233-7)

# Supplementary material:

# Vapor sorption

Moisture uptake of the prepared filaments presented as mass change (%) at different relative humidity (RH). Data obtained from dynamic vapor sorption analysis. Data for 90% RH missing for formulation 2 and 12, due to technical issues during the measurement. Dm = mass change.


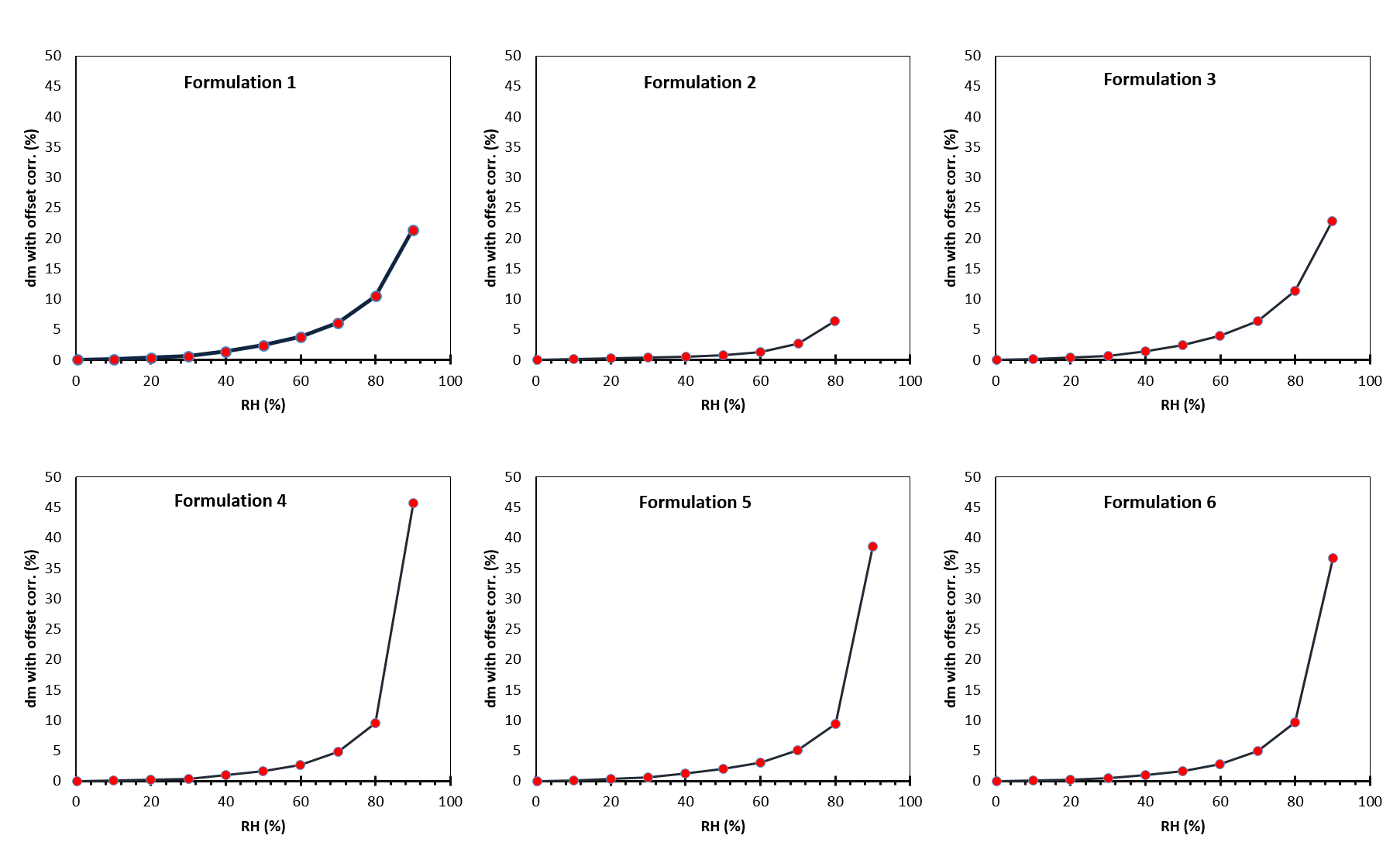


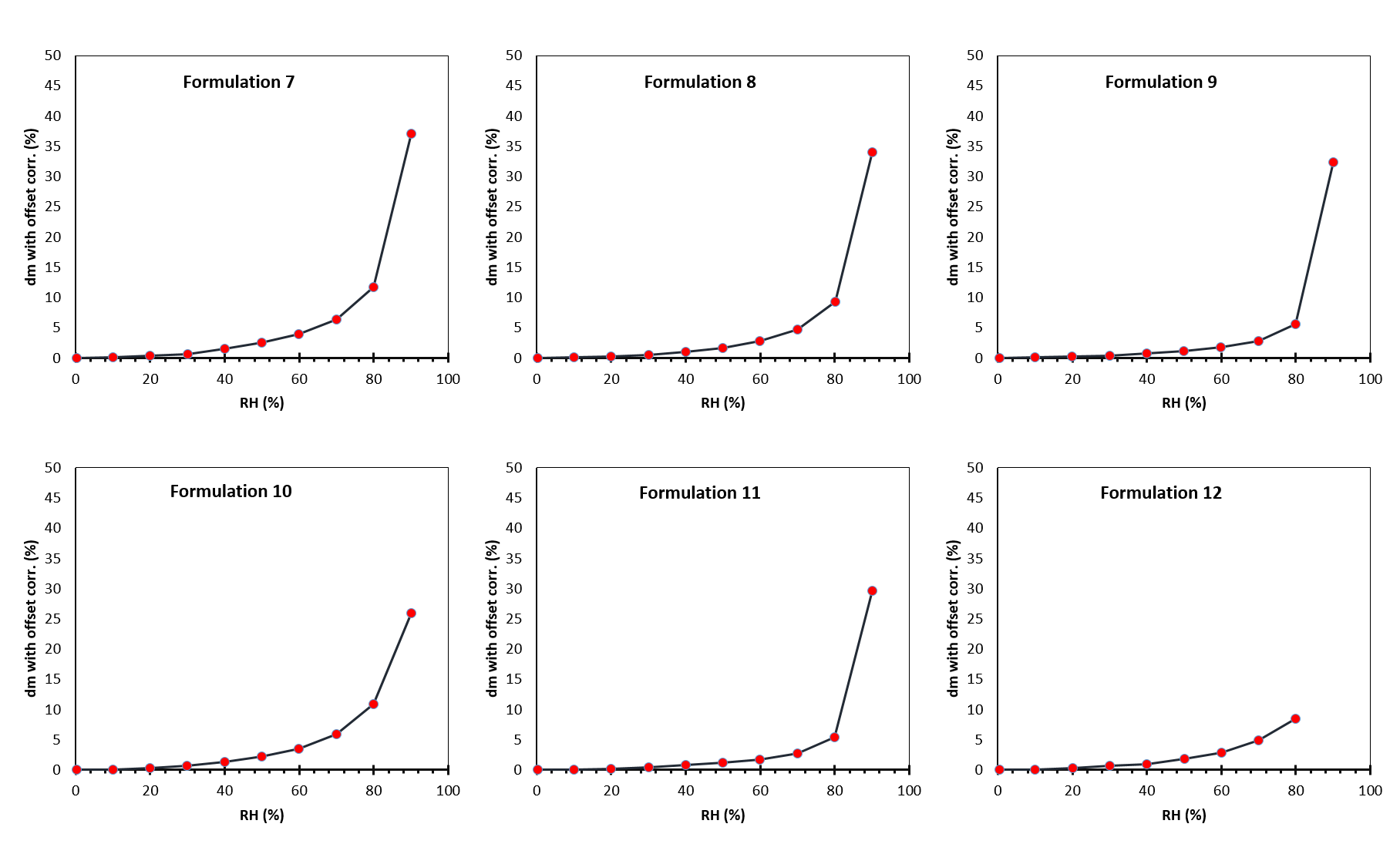


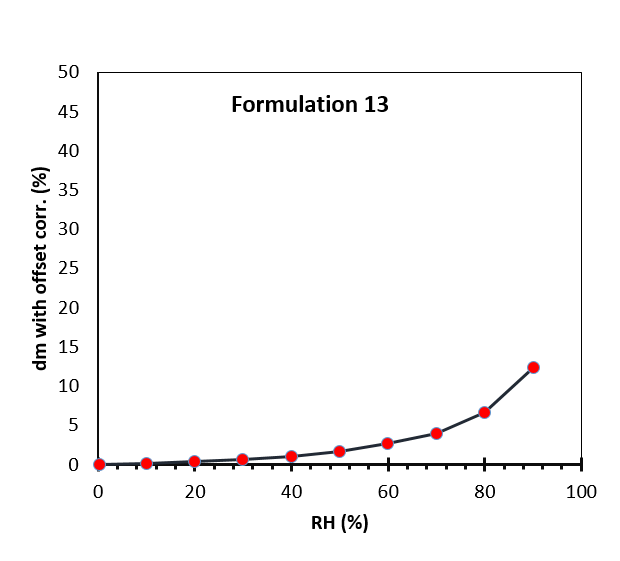


DSC

Heat-cool-heat thermograms for the raw materials, physical mixtures (PM), hot-melt extruded material (HME) and subsequently 3D-printed tablets (3DP).


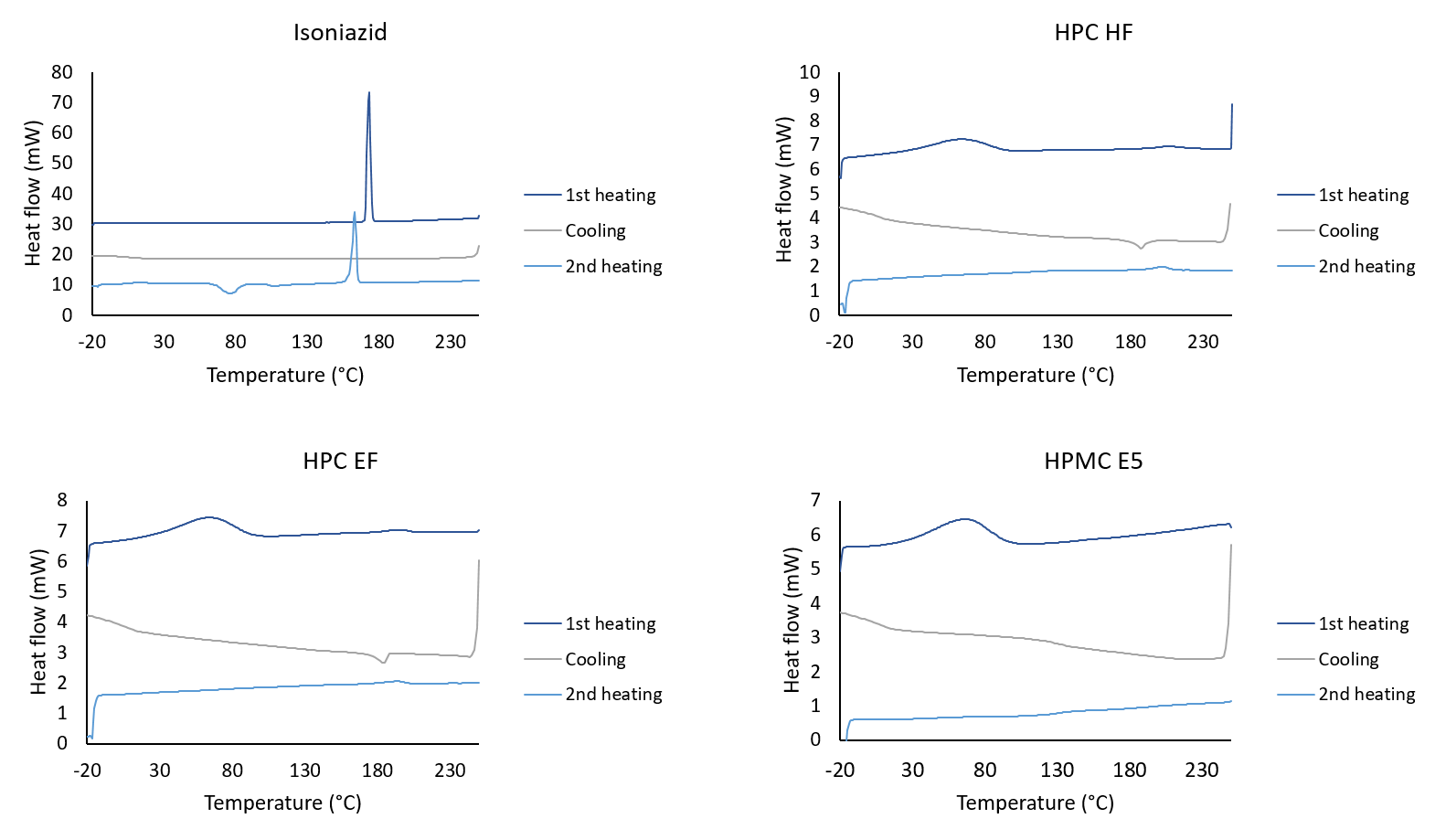


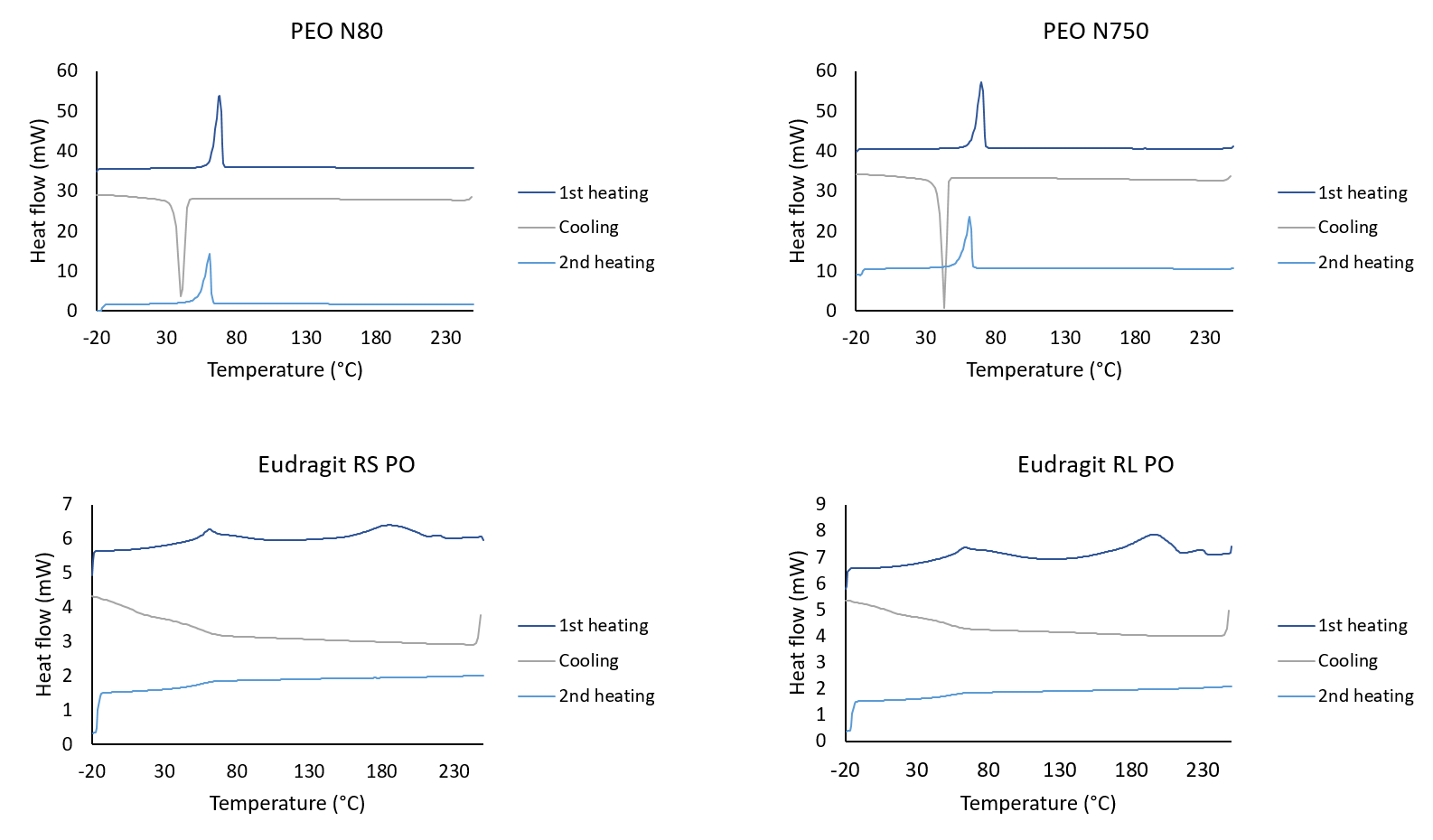


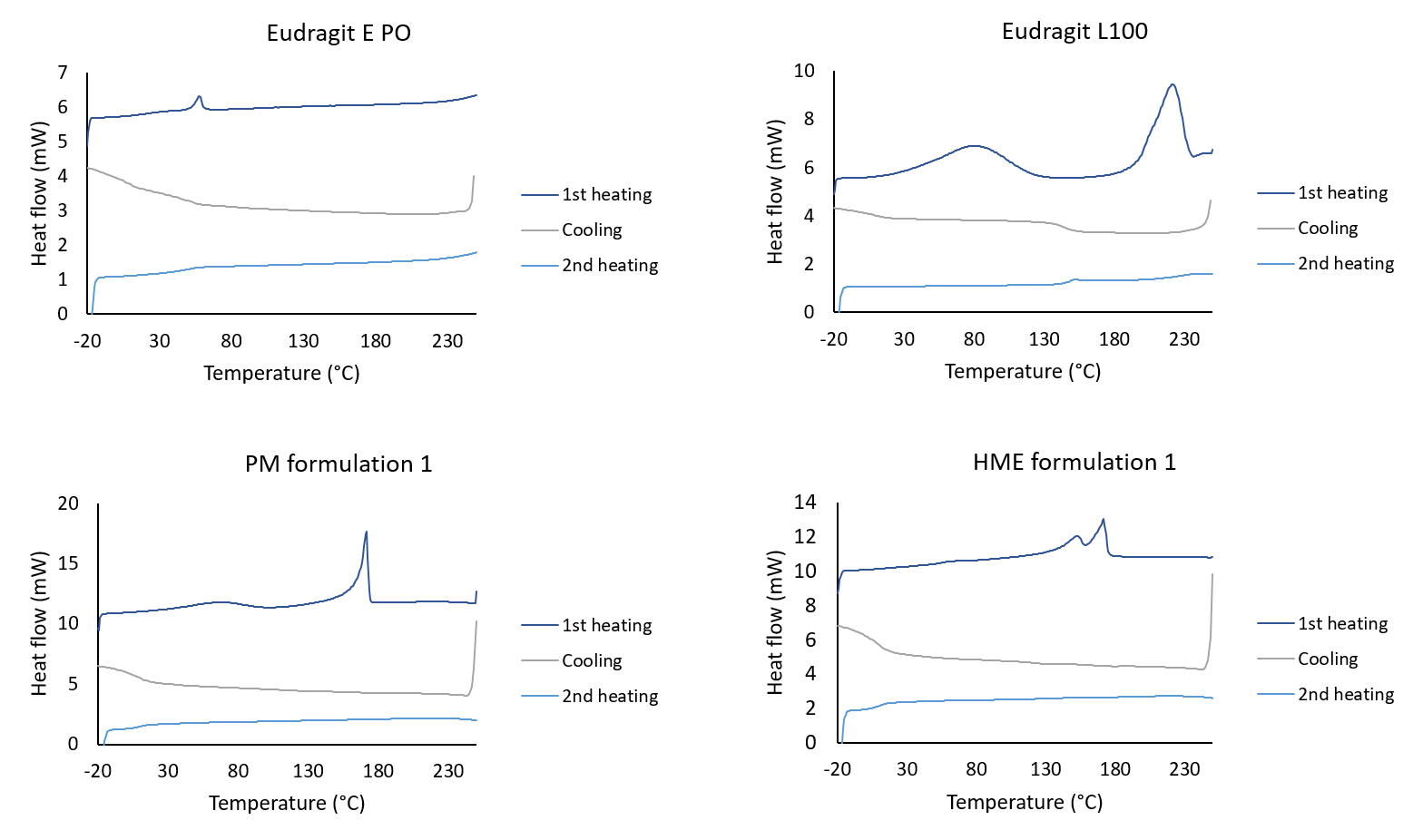


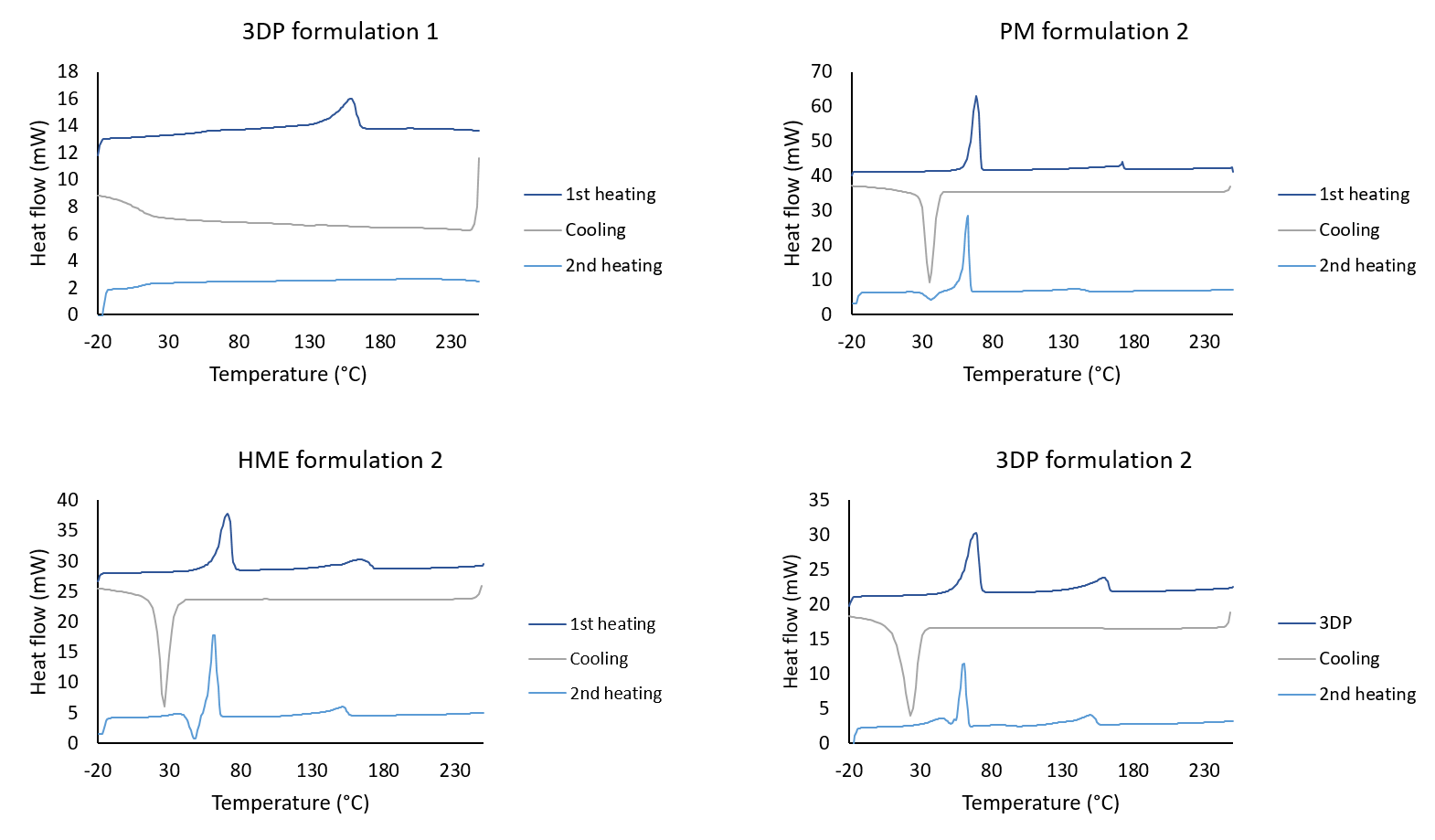


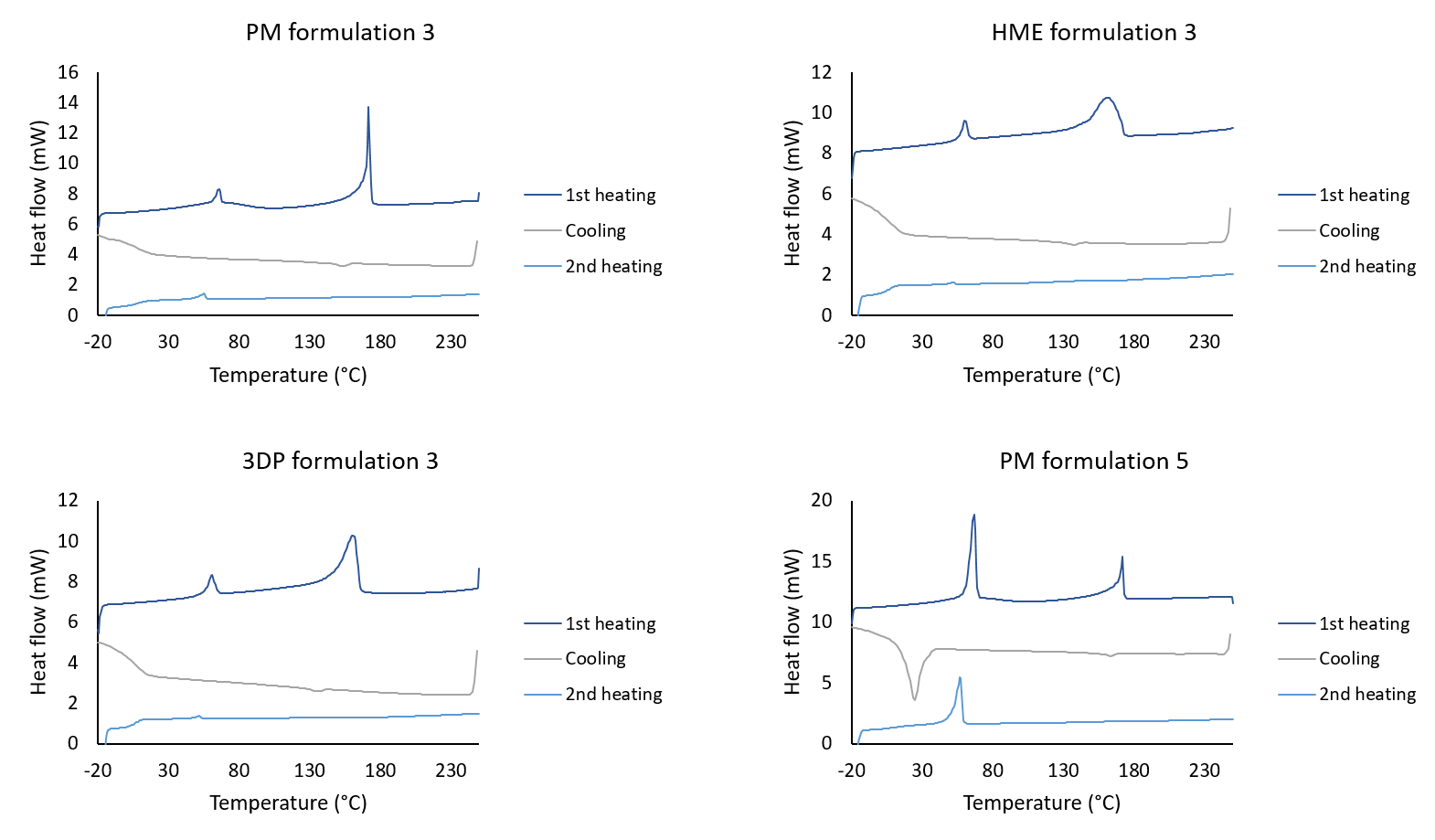


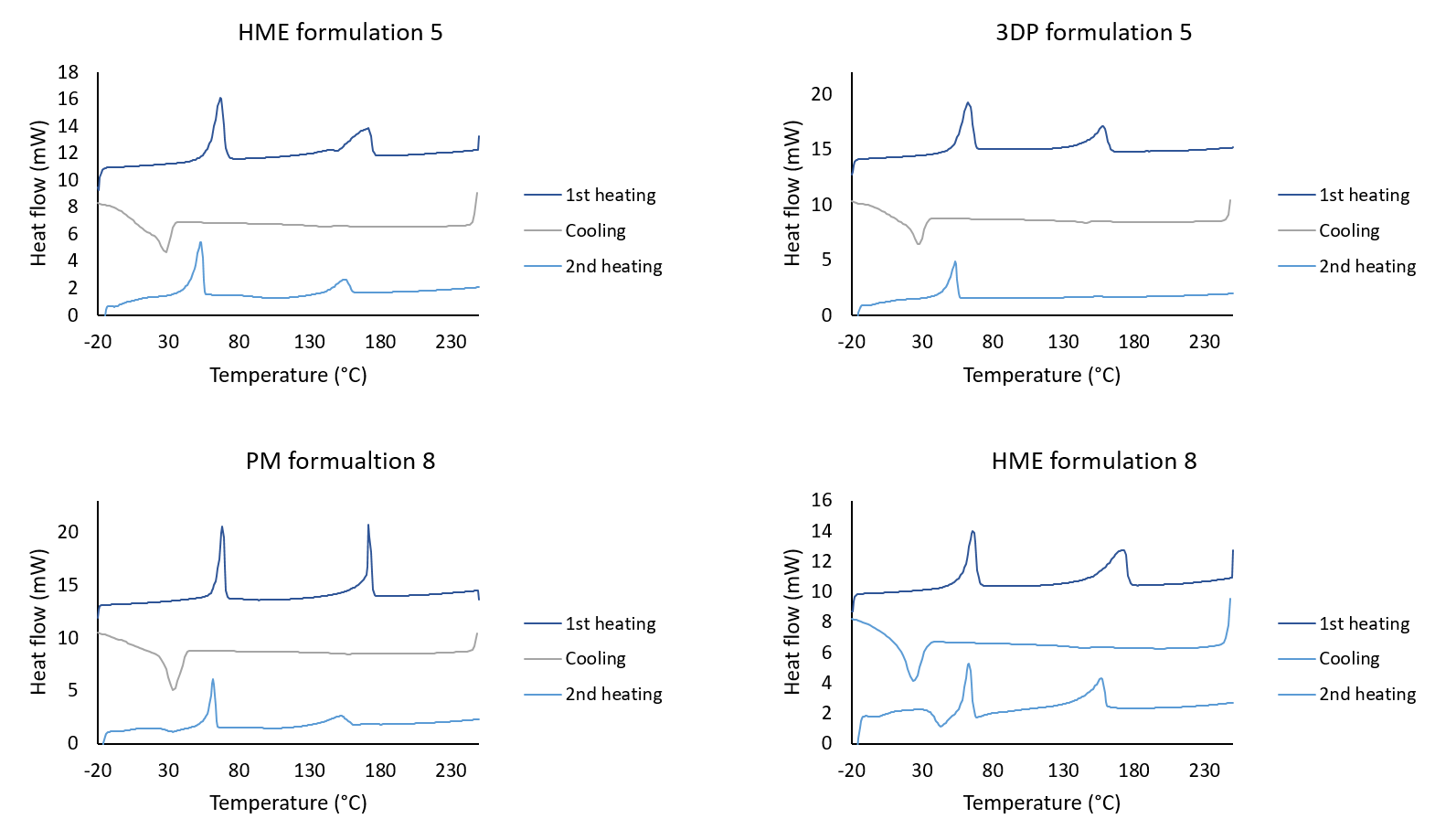


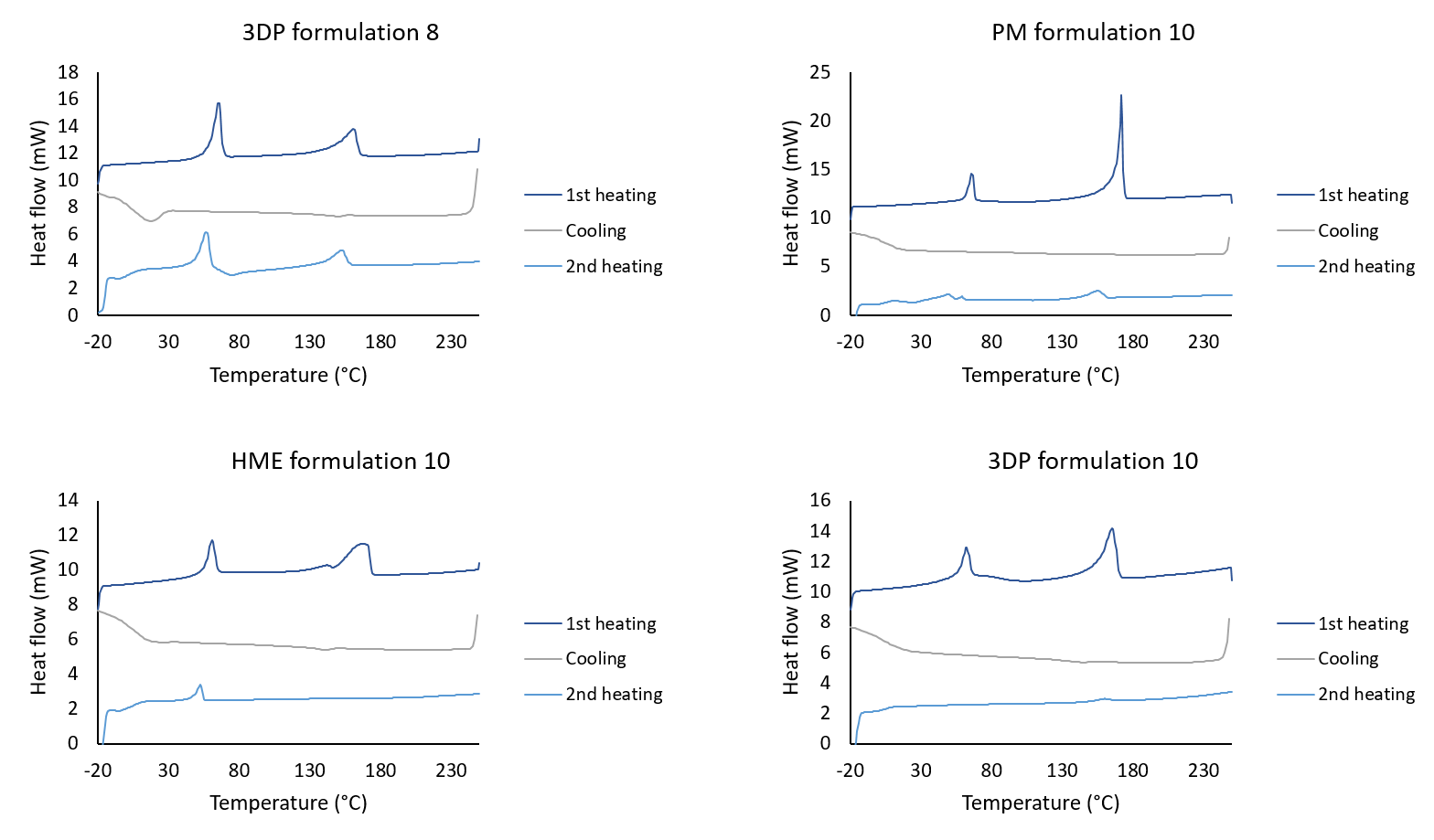


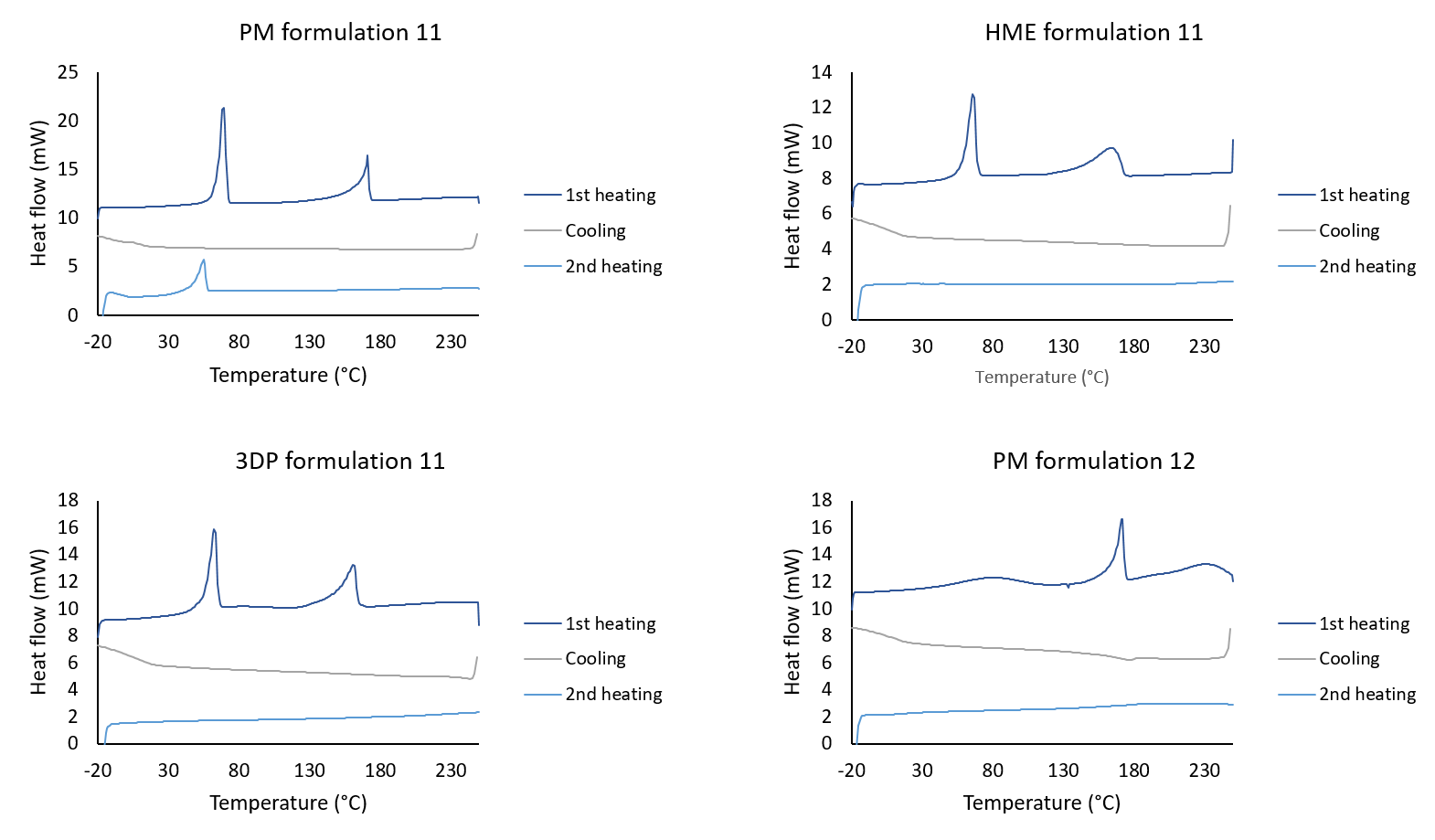


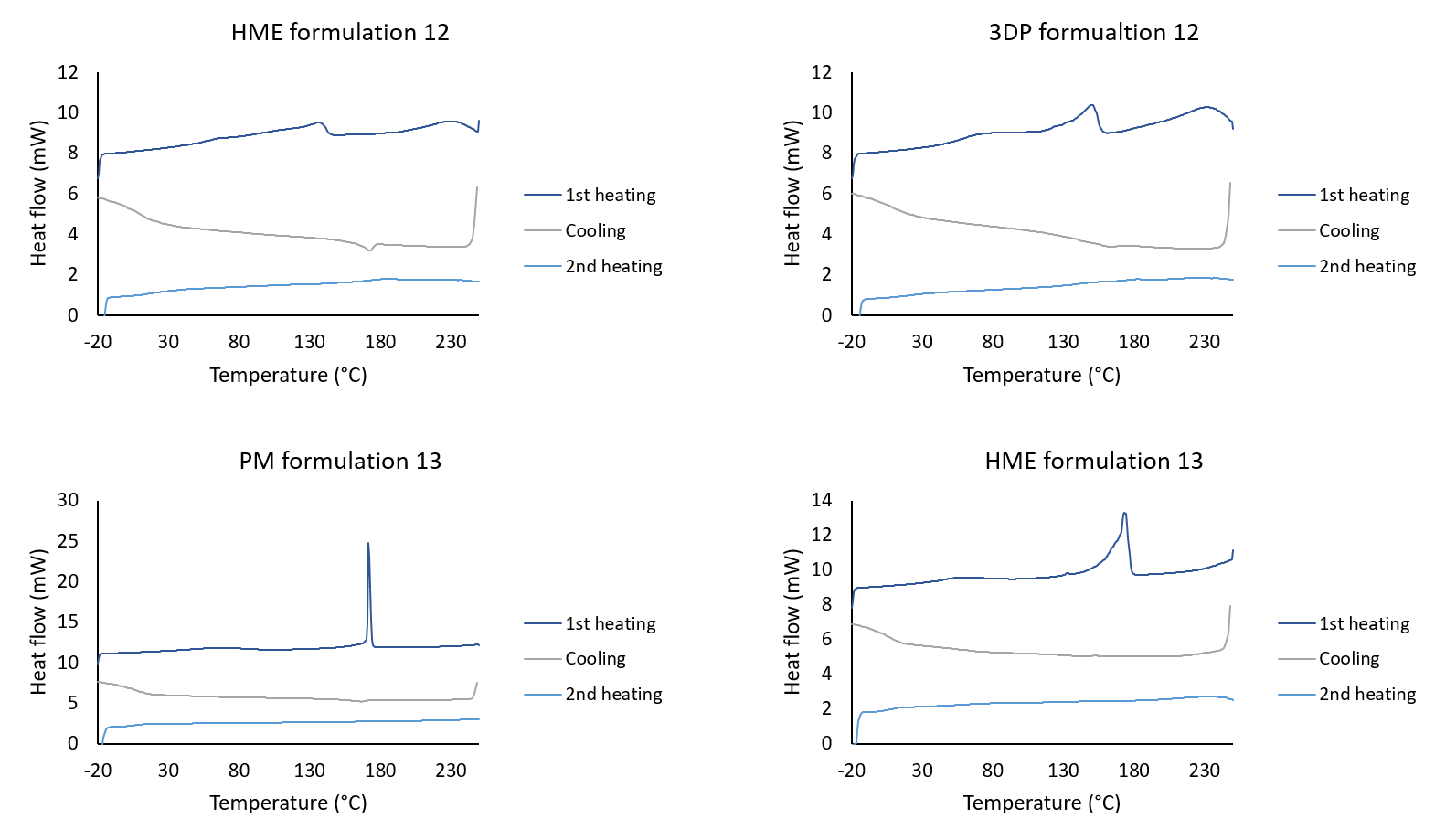


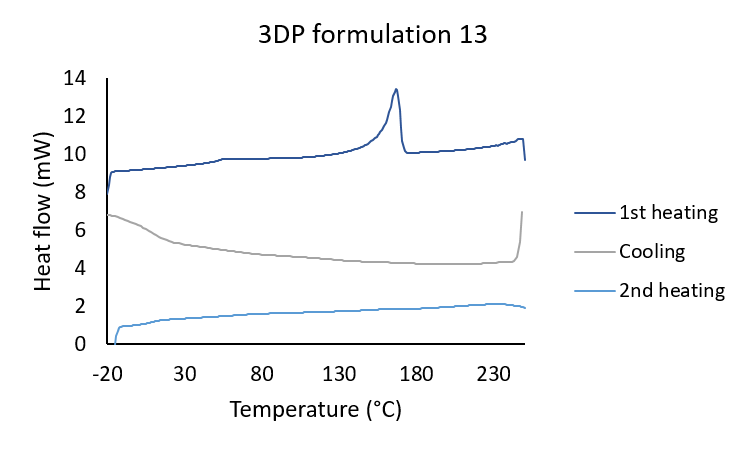


# Dissolution

Drug release for hot-melt extruded filaments (HME filament), 3D-printed filaments (3DP filament) with the same dimensions as the HME filament, 3D-printed tablets (3DP tablet) of different sizes and infill levels (mean ± SD, n = 3). Note that the time scale is different for the different graphs to better visualize differences. Filaments presented in grey and 3D-printed tablets in blue.


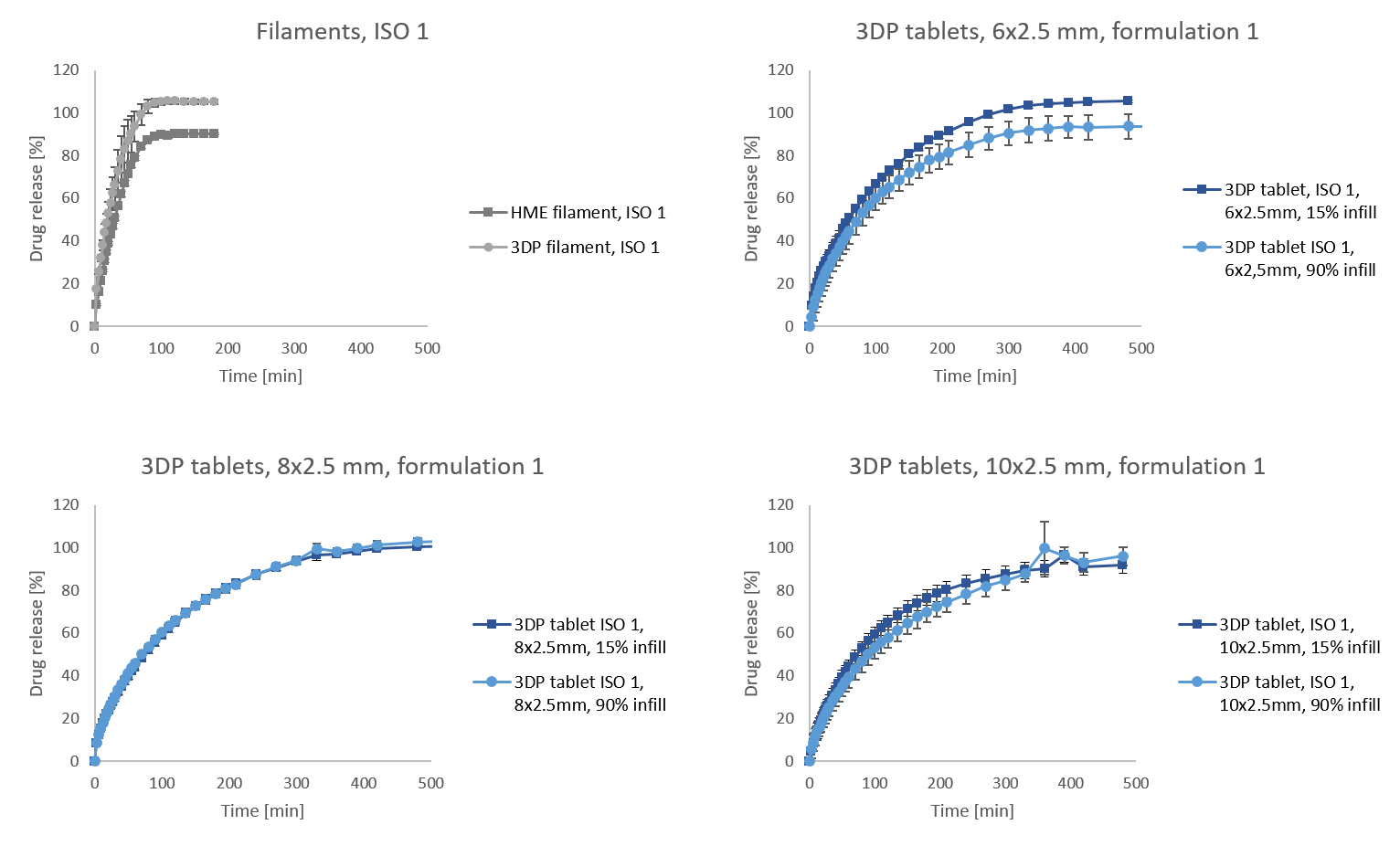


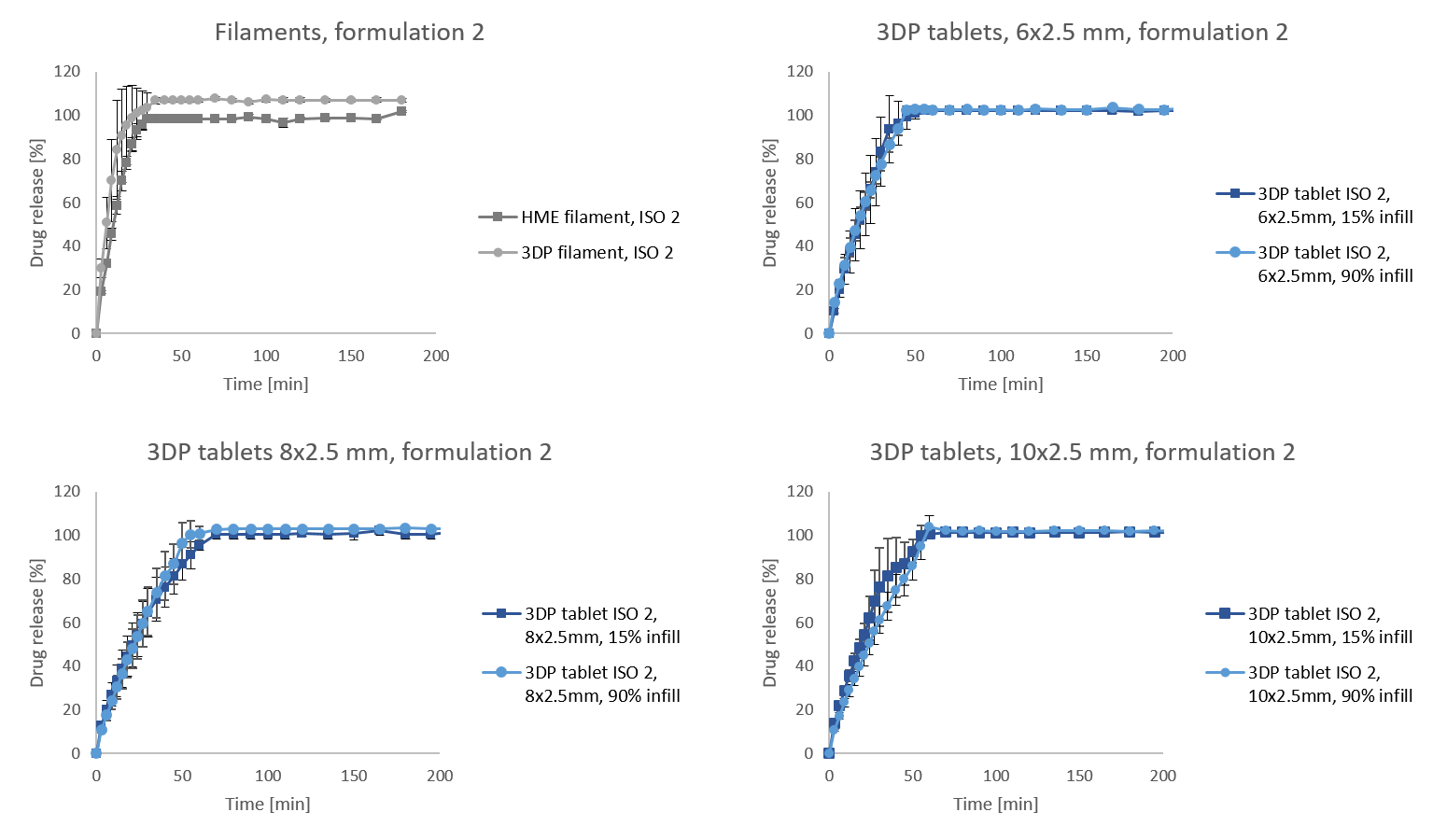


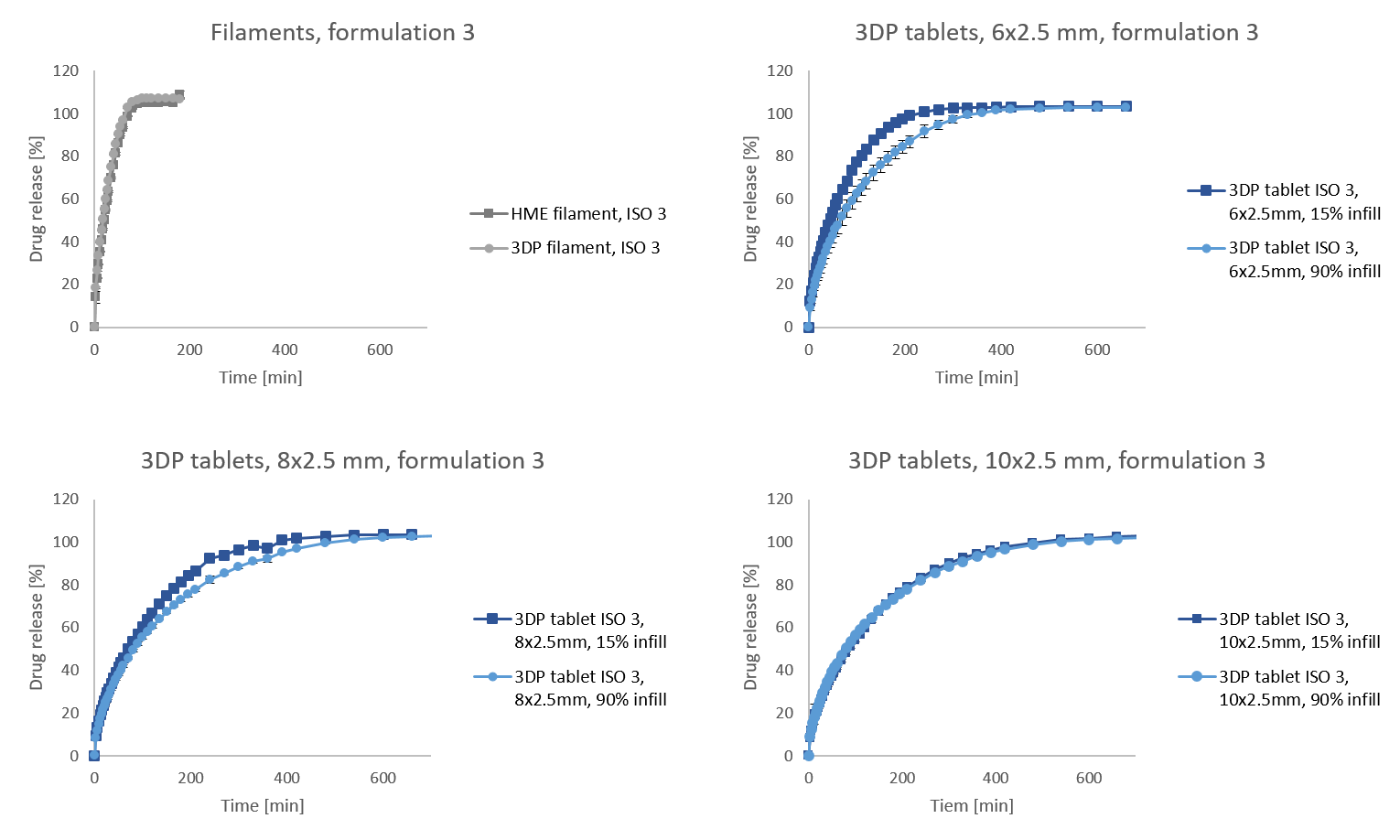


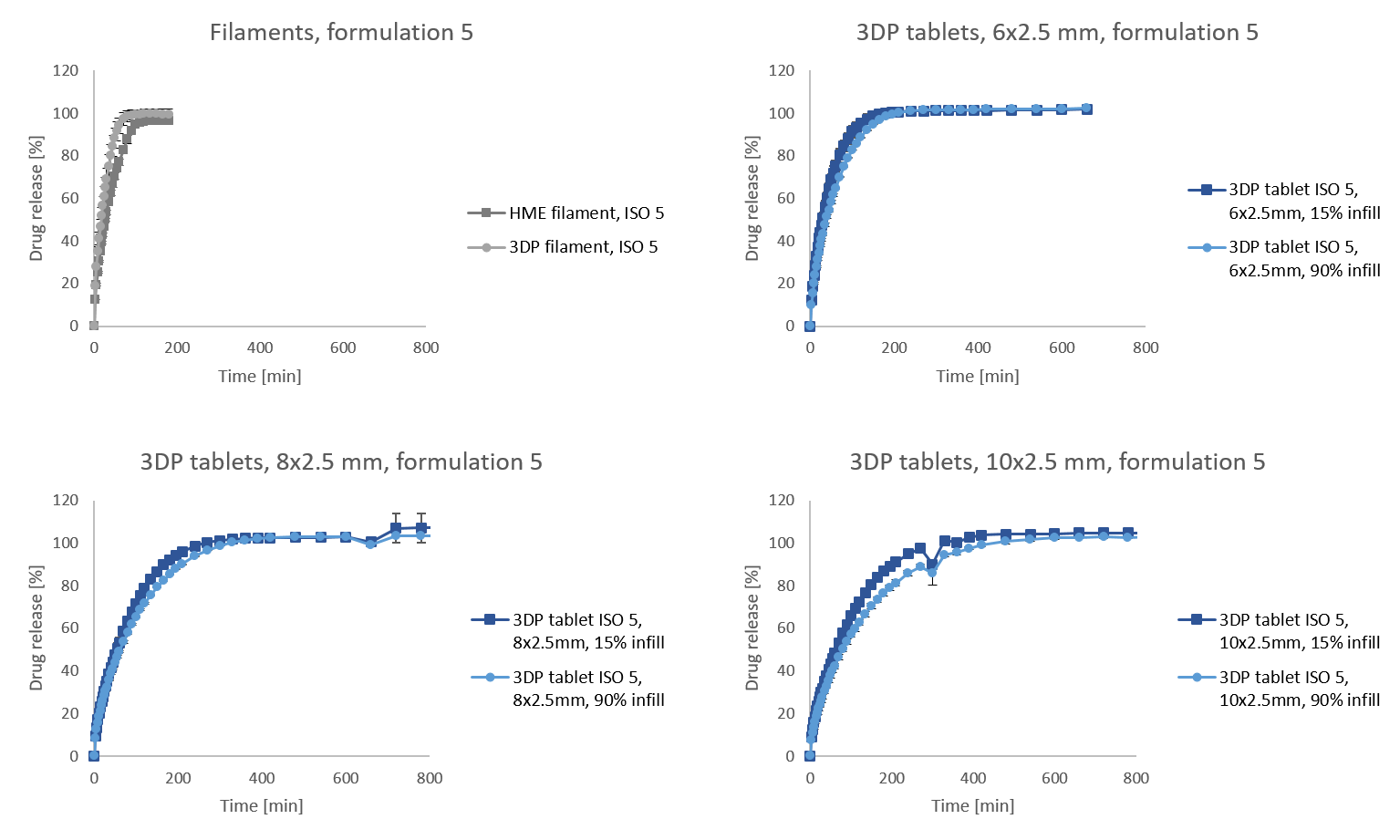


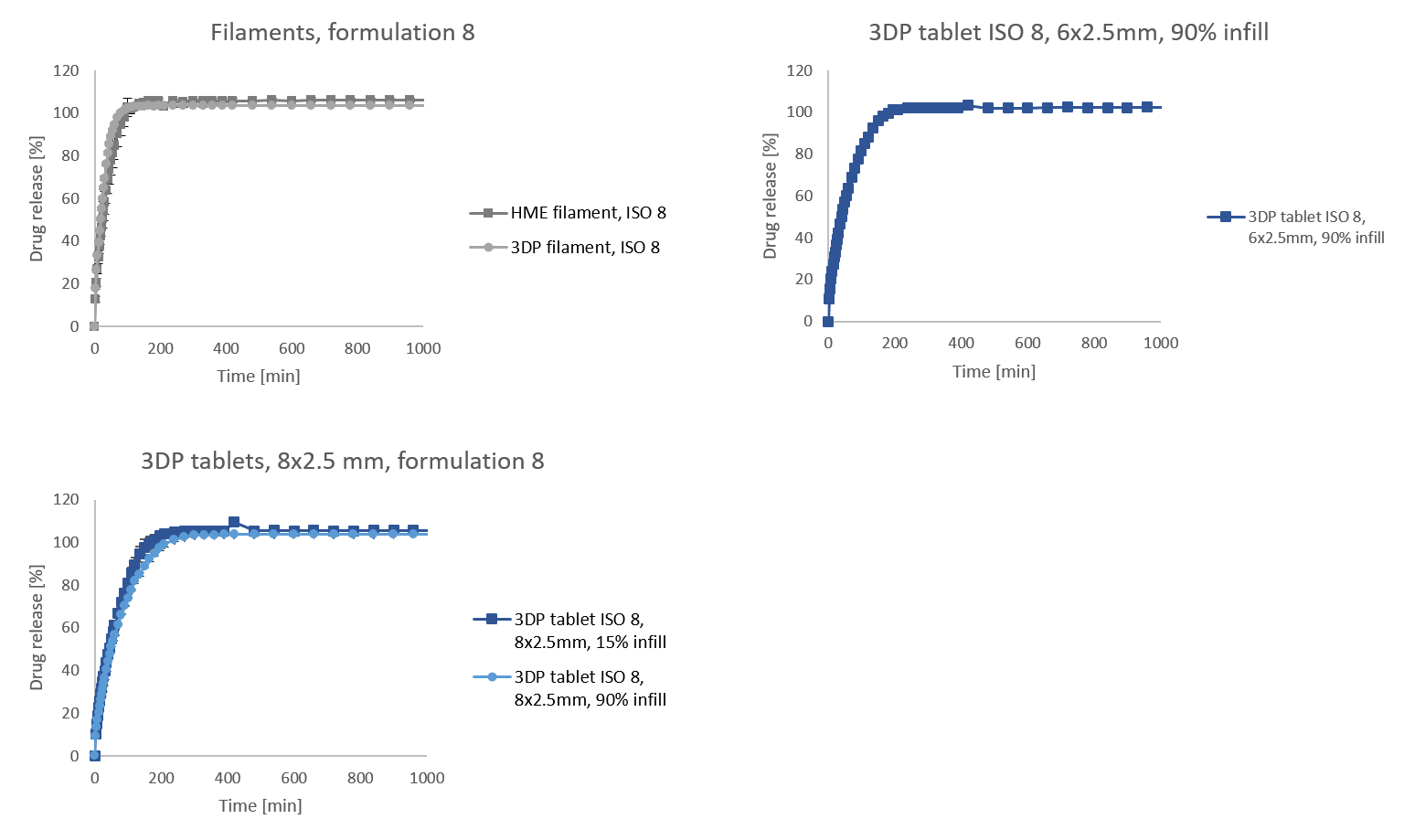


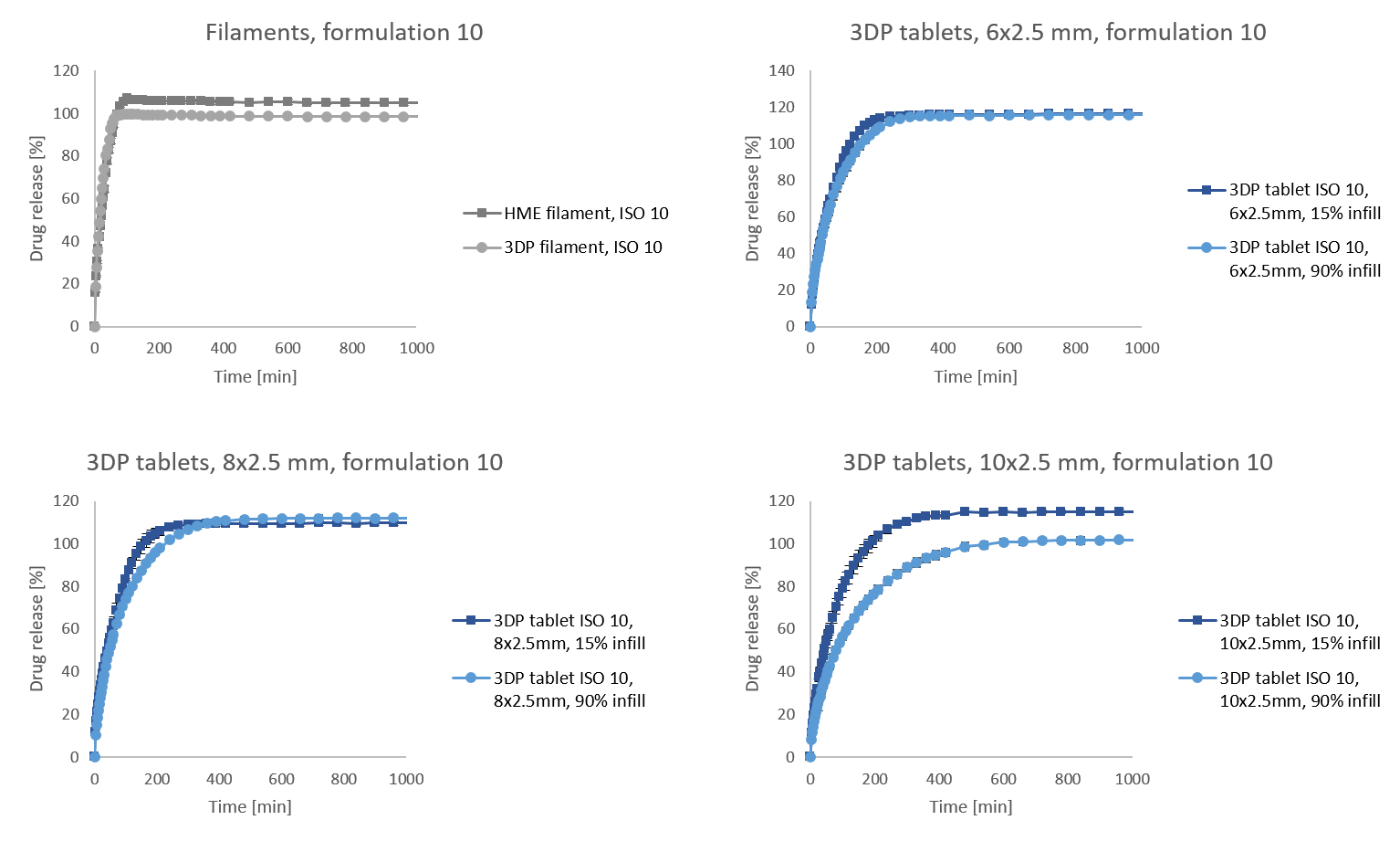


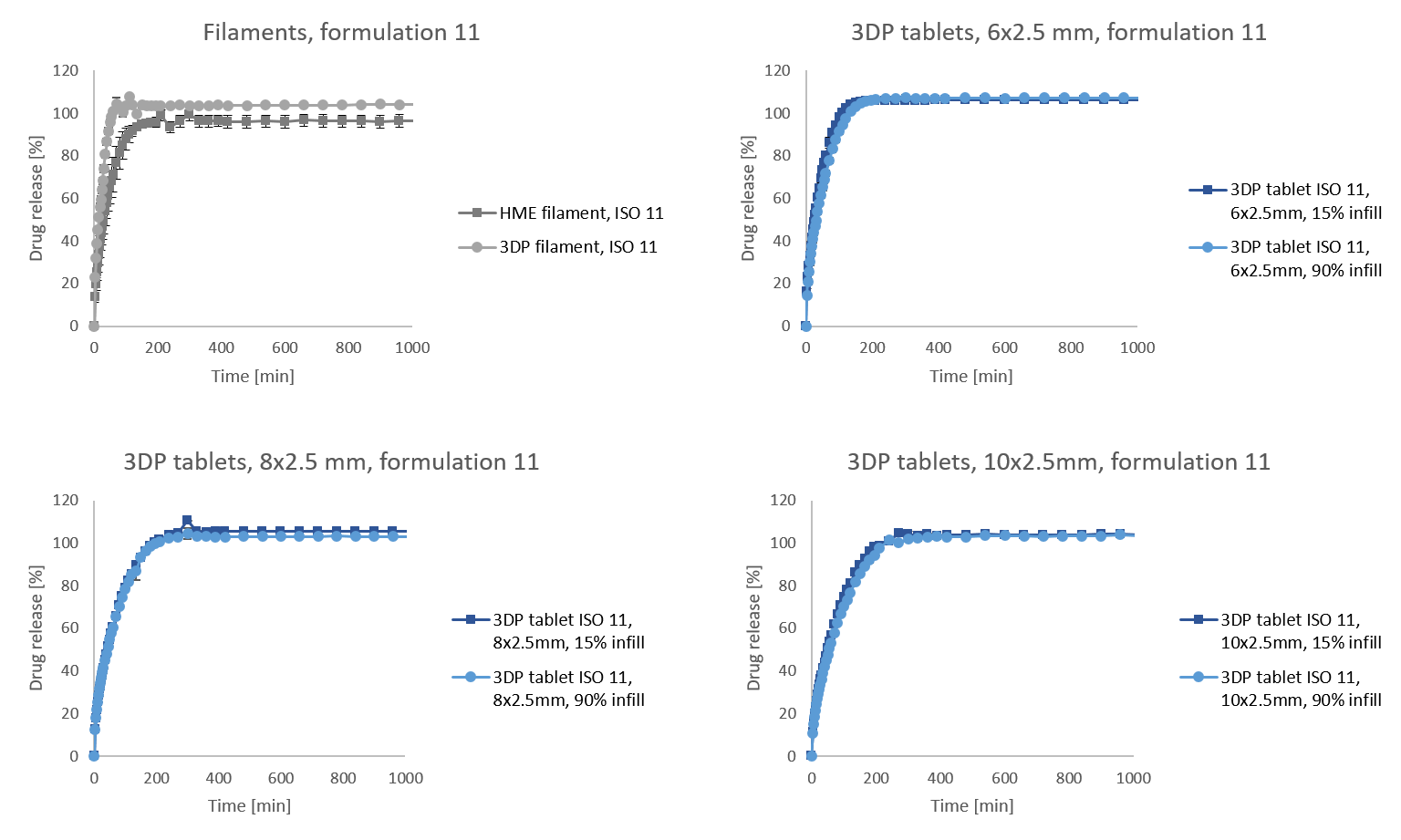


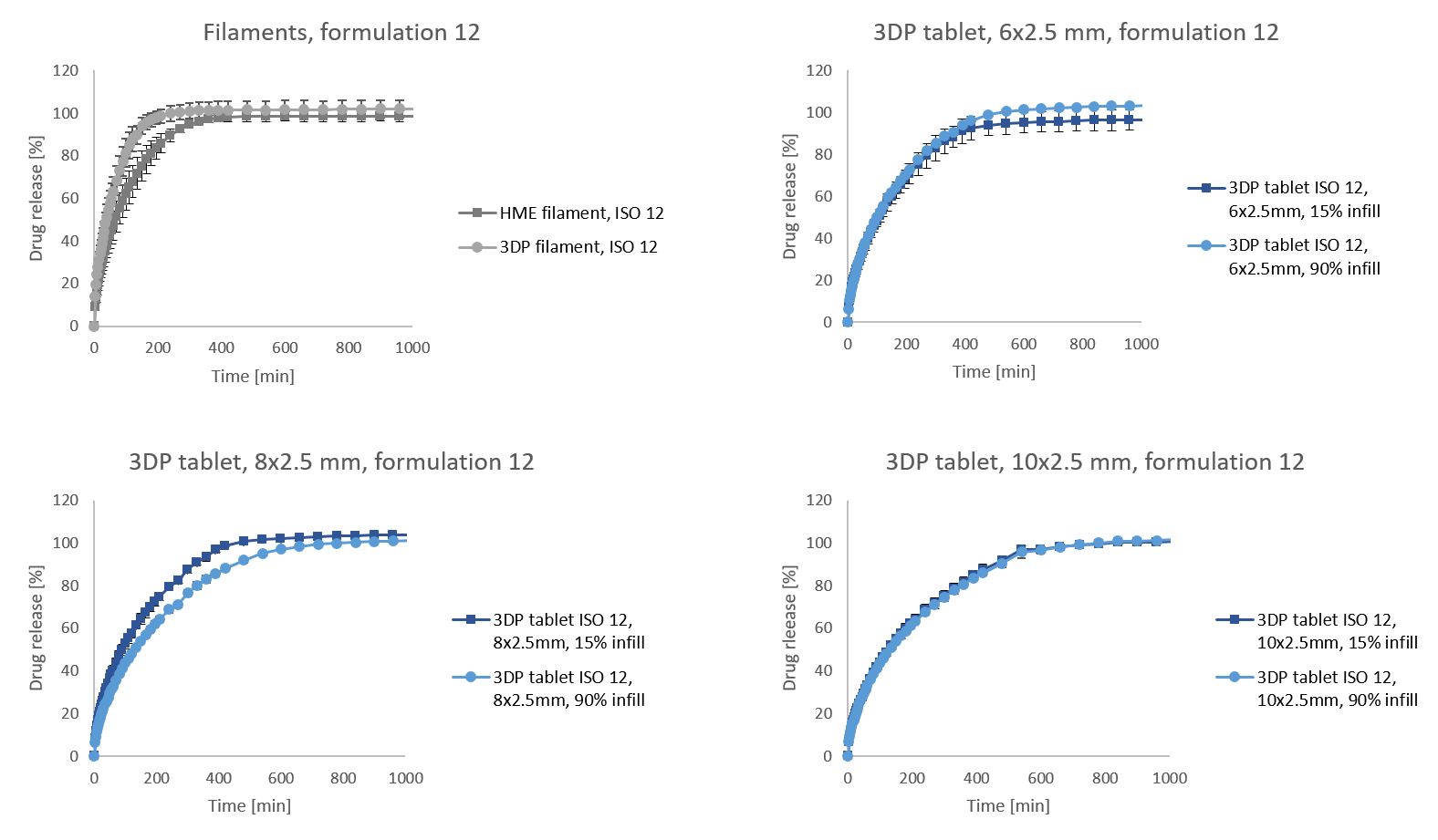


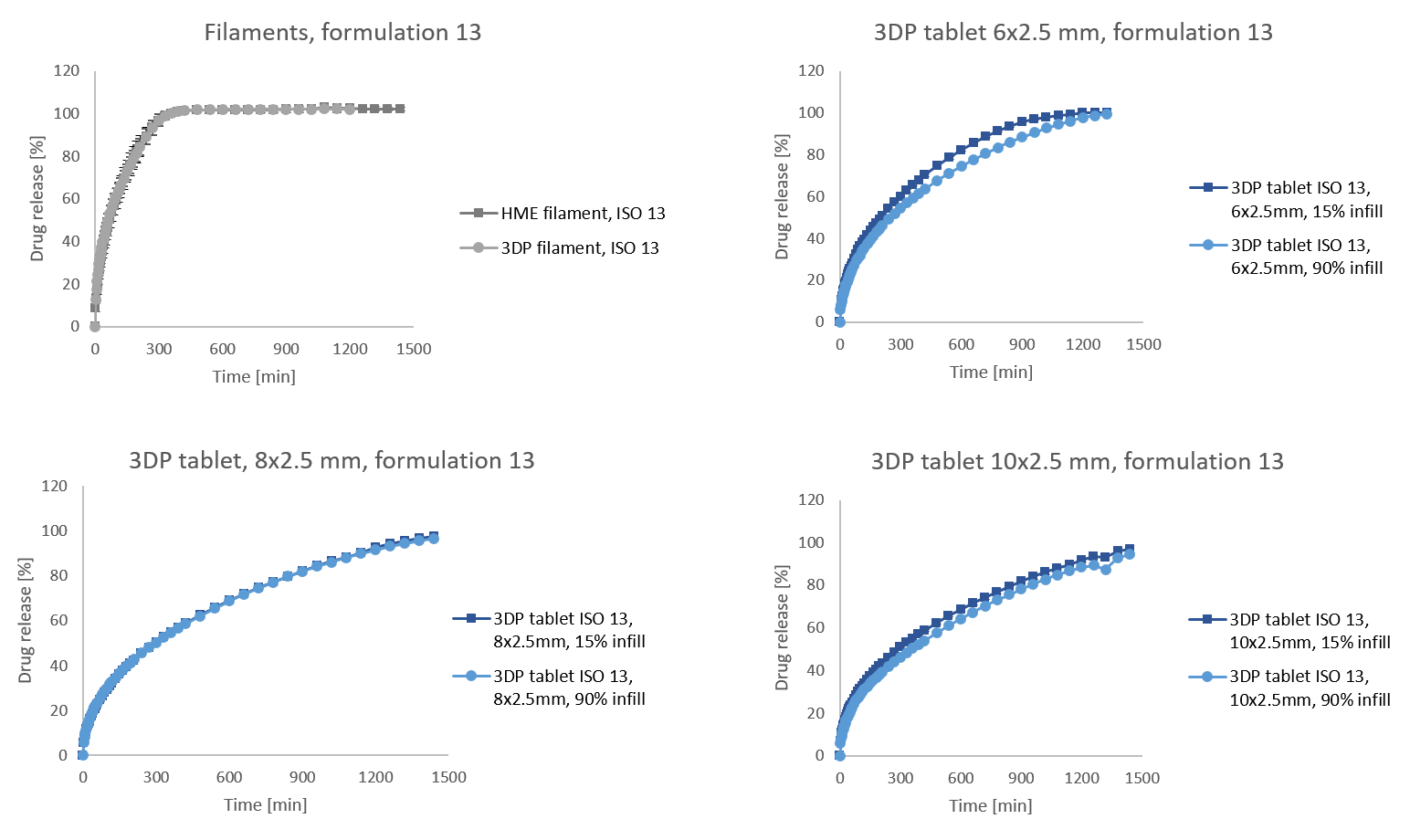

Supplement: Supplementary file 1 — (DOCX 3477 kb) [file 12249_2018_1233_MOESM1_ESM.docx]
